# Impaired autophagic degradation of lncRNA ARHGAP5-AS1 promotes chemoresistance in gastric cancer

**DOI:** 10.1038/s41419-019-1585-2

**Published:** 2019-05-16

**Authors:** Liyuan Zhu, Yiran Zhu, Shuting Han, Miaoqin Chen, Ping Song, Dongjun Dai, Wenxia Xu, Tingting Jiang, Lifeng Feng, Vivian Y. Shin, Xian Wang, Hongchuan Jin

**Affiliations:** 1 Laboratory of Cancer Biology, Key Laboratory of Biotherapy of Zhejiang Province, Sir Run Run Shaw Hospital, Medical School of Zhejiang University, Hangzhou, China; 2 Department of Medical Oncology, Sir Run Run Shaw Hospital, Medical School of Zhejiang University, Hangzhou, China; 3 Department of Surgery, the University of Hong Kong, Hong Kong SAR, China

**Keywords:** Cancer therapy, Cancer therapy

## Abstract

Chemoresistance remains the uppermost disincentive for cancer treatment on account of many genetic and epigenetic alterations. Long non-coding RNAs (lncRNAs) are emerging players in promoting cancer initiation and progression. However, the regulation and function in chemoresistance are largely unknown. Herein, we identified ARHGAP5-AS1 as a lncRNA upregulated in chemoresistant gastric cancer cells and its knockdown reversed chemoresistance. Meanwhile, high ARHGAP5-AS1 expression was associated with poor prognosis of gastric cancer patients. Intriguingly, its abundance is affected by autophagy and SQSTM1 is responsible for transporting ARHGAP5-AS1 to autophagosomes. Inhibition of autophagy in chemoresistant cells, thus, resulted in the upregulation of ARHGAP5-AS1. In turn, it activated the transcription of ARHGAP5 in the nucleus by directly interacting with ARHGAP5 promoter. Interestingly, ARHGAP5-AS1 also stabilized ARHGAP5 mRNA in the cytoplasm by recruiting METTL3 to stimulate m^6^A modification of ARHGAP5 mRNA. As a result, ARHGAP5 was upregulated to promote chemoresistance and its upregulation was also associated with poor prognosis in gastric cancer. In summary, impaired autophagic degradation of lncRNA ARHGAP5-AS1 in chemoresistant cancer cells promoted chemoresistance. It can activate the transcription of ARHGAP5 in the nucleus and stimulate m^6^A modification of ARHGAP5 mRNA to stabilize ARHGAP5 mRNA in the cytoplasm by recruiting METTL3. Therefore, targeting ARHGAP5-AS1/ARHGAP5 axis might be a promising strategy to overcome chemoresistance in gastric cancer.

## Introduction

Despite of significant advances, cancers such as gastric cancer remains one of the uppermost causes of mortality^1,2^. At present, the general treatment of cancers include neoadjuvant chemotherapy and adjuvant chemotherapy, before and after surgery, respectively^3^. However, chemoresistance will eventually appear to confer the treatment failure. Chemoresistance usually display resistance to many chemotherapeutic drugs through various mechanisms including both genetic and epigenetic changes, alterations in multiple signaling pathway or cell metabolism^4^.

Autophagy, an evolutionarily conserved process for the intracellular recycling and degradation, plays a pivotal role in molecular, cellular, and tissue homeostasis by coordinating metabolism and signaling pathways^5–7^. Recently, autophagy is emerging as a preeminent factor to modulate tumorigenesis and cancer therapy in response to metabolic stress, cellular damage, and detoxication^5,7^. Autophagy might promote chemoresistance by enabling cancer cell survival under chemotherapeutic stresses^8,9^. However, it can also promote cell apoptosis, thus enhancing chemosensitivity^10,11^. Therefore, the regulation and function of autophagy in drug response and chemoresistance development warrant further investigations.

Discovery of enormous LncRNAs in diverse kinds upend the traditional insight about genome organization. This “dark matter” gradually demonstrated the “bright side” with multiple functions in cell fate determination^12–14^. Mechanically, the localization of lncRNAs vary from chromatin to subnuclear domains or cytoplasm, participating in the regulation of chromatin remodeling, gene transcription, RNA splicing, microRNA interaction, RNA stability or transportation and protein functions^15,16^. Among them, natural antisense transcripts (NATs) are simply complementary to their RNA transcripts in opposite strand and non-coding in majority. NATs could involve in regulating gene expression or stability through DNA–RNA, RNA–RNA, or protein–RNA interactions and the dysregulation is implicated in many pathogeneses including tumorigeneses^17,18^. Historically, accumulating evidence indicated that chemoresistance was associated with the alteration in expression of some lncRNAs, such as UCA1^19^, linc-ROR^20^, and GAS5^21,22^.

In this study, we identified a chemoresistance-promoting NAT named ARHGAP5-AS1. It was upregulated in chemoresistant gastric cancer cells, resulting from impaired autophagic degradation. Intriguingly, ARHGAP5-AS1 increased the expression of its target gene ARHGAP5 to promote chemoresistance. Inhibiting either ARHGAP5-AS1 or ARHGAP5 succeeded to reverse chemoresistance.

## Materials and methods

### Cell culture, antibodies, and chemicals

Human gastric cancer cell lines SGC7901 and BGC823 were all purchased from the Type Culture Collection of the Chinese Academy of Sciences (Shanghai, China). Multidrug-resistant cells SGC-R and BGC-R were induced from SGC7901 and BGC823, respectively^23^. Cells were all cultured in RPMI-1640 medium (Invitrogen) medium supplemented with 10% FBS and 100 U/mL penicillin–streptomycin. All the antibodies used in our study were listed as: anti-cleaved PARP1 (CST, 9541-s); anti-cleaved Caspase3 (CST, 9661S); anti-β-actin (CST, 8457); anti-RhoGAP (ARHGAP5) (Abcam, ab32328); anti-LC3B (Novus, NB100-2220); anti-SQSTM1 (MBL, PM045); anti-FLAG (Sigma, F1804-1); anti-GAPDH (Abcam, ab75834); anti-H3 (Abcam, ab1791); anti-HuR (Abcam, AB200342); anti-METTL3 (Abclonal, a8370); anti-METTL14 (Abclonal, a8530), and anti-WTAP (Abcam, ab195380). The chemicals used in this study include chloroquine (CQ) (C6628), cisplatin (DDP, Selleck), rapamycin (Sigma-Aldrich), actinomycin D (Abcam, ab141058), doxorubicin hydrochloride (ADM, Main Luck), and 5-fluoracil (5-FU, KingYork).

### RNA extraction and quantitative real-time PCR

Total RNAs were isolated using the Trizol reagent (Invitrogen) and concentrations were quantified using NanoDrop 2000 (Wilmington, DE, USA), followed with DNase I digestion and reverse transcribed by random primers to generate cDNA templates strictly according to the manufacturer’s instructions (Thermofisher Scientific Inc., Shanghai, China). Nuclear or Cytoplasmic RNA were isolated using the Thermo Scientific™ NE-PER™ Nuclear or Cytoplasmic RNA Purification Kit according to manufacturer’s protocols. Real-time quantitative PCR was performed using SYBR Green Master Mix Kit and Light Cycler 480 II system (Roche, Shanghai, China). To determine relative gene expression, RNA integrity was normalized to internal control β-actin or 18S. All primer sequences used for PCR are listed in Supplemental Table 1.

### Plasmids, siRNAs, and transfection

The full length of ARHGAP5-AS1 was cloned to pcDNA3.1 vector. Various truncation segments of SQSTM1 was cloned to EX05-flag vector. Plasmids for preparing probe were all segment-pGEM-T. All of the plasmids were purified using the EndoFree Plasmid Maxi Kit (QIAGEN) and transfected into cells using X-tremeGENE HP DNA Transfection Reagent (Roche Applied Science, Shanghai, China). Specific siRNAs were designed and synthesized by Gene Pharma Company (Shanghai, China). SiRNAs were transfected using Lipofectamine^TM^ RNAiMAX transfection reagent (Thermo Fisher Scientific) at a final concentration of 100–200 nM. All siRNAs sequences used for knockdown are listed in Supplemental Table 1.

### Cell viability and apoptosis assay

Cell viability was measured using the 3-(4, 5-dimethylthiazol-2-yl)-5-(3-carboxymethoxyphenyl)-2-(4-sulfophenyl)-2H-tetrazolium (MTS) standard method. For apoptosis analysis, the cells were harvested and assessed for apoptosis using Annexin V-FITC-PI dual-staining kit (556547; BD Biosciences, USA) by flow cytometer.

### ADM concentration determination

About 5 × 10^5^ cells were seeded overnight in six-well plate and transfected with given siRNAs or plasmids for 48 h, and incubated with doxorubicin hydrochloride (ADM, 5 μg/mL) at 37 °C for 2 h. After collecting cells in a single cell suspension, the raw fluorescence intensity of single cells was measured using flow cytometry in FL2 channel.

### Autophagy induction

Autophagy was effectively induced using EBSS treatment for 2 or 4 h, amino acid deprivation medium (NaCl 140 mM, CaCl_2_ 1 nM, MgCl_2_·6H_2_O 1 mM, d-glucose 5 mM, hepes 20 mM, BSA 1% in 1 × PBS) treatment for 8 or 12 h, or rapamycin (50 or 200 nM) treatment for 48 h.

### RNA half-life assay

Cells were treated with given siRNAs or plasmids or chemicals for 48 h and treated with Actinomycin D (Act D; 5 μg/mL) to block the synthesis of new RNA. Cells were then harvested to extract with total RNA at 0, 2, 4, 6, 8, 12 h after Act D addition for quantitative RT-PCR.

### RNA immunoprecipitation (RIP) assay

RIP was performed using Magna RIP^TM^ RNA-Binding Protein Immunoprecipitation Kit (Millipore, No.17-700). Briefly, 1 × 10^7^ cells after the given treatment were lysed in 100 μL RIP lysis buffer with Protease Inhibitor and RNase Inhibitor, and immunoprecipitated with antibodies of interest and protein G magnetic beads for overnight at 4 °C, followed by six washes in Washing Buffer and protein digestion at 55 °C. Total RNA was isolated from the aqueous after digestion and subjected to RT-PCR analysis for quantification.

### Co-localization assay

The assay mainly combined RNA florescence in situ hybridization (FISH) (LGC Science Ltd.) and immunofluorescence (IFC) with some modifications. Firstly, cells in 12-well plate were fixed using fixation buffer and incubated with the Stellaris RNA FISH probe (LGC) in hybridization buffer for at least 4 h at 37 °C after using 0.2% Triton X-100 for 20 min to permeate the cells. After washing with Wash Buffer A, the cells were blocked with 3% BSA for 1 h at RT and incubated with anti-SQSTM1 or anti-LC3B antibody at 4 °C overnight. After washing with PBST three times, the cells were incubated with secondary fluorescent antibodies for 1 h at RT before proceeding to imaging.

### RNA pull down assay

Firstly, Streptavidin Sepharose (GE Healthcare) was pretreated using RNA-binding buffer (50 mmol/L KCl, 1.5 mmol/L MgCl_2_, 10 mmol/L HEPES (PH 7.5), 0.5% NP40, 2 mmol/L DTT, 1 mmol/L EDTA, 100 U/mL RNase Inhibitor, Protease Inhibitor, 100 μg/mL tRNA, and 400 μmol/L Vanady ribonucleoside complexes). RNA–protein complex was formed by incubating 1–2 μg biotin-labeled probe with cell lysates at 30 °C for 30 min. After incubating pretreated Streptavidin Sepharose at RT for 50 min, RNA–protein mixture was precipitated and extracted proteins for Western blotting with 20 μL 6×Loading buffer after six times strictly washing with RNA Washing Buffer (50 mmol/L KCl, 1.5 mmol/L MgCl_2_, 10 mmol/L HEPES (PH 7.5), 0.5% NP40).

### Biotin pull down assay

The detailed procedures were according to previously described^24^. In brief, cells were transfected with ARHGAP5-AS1 biotinylated probes for 48 h and resuspended using lysis buffer (20 mM Tris, pH 7.5, 200 mM NaCl, 2.5 mM MgCl_2_, 0.05% Igepal, 60 U/mL, Superase-In (Takara), 1 mM DTT, protease inhibitors (Roche)). 50 μL lysates were used for input control. Lysates were incubated with prepared streptaviden beads (GE Healthcare). RNase-free bovine serum albumin (BSA) and yeast tRNA (both from Sigma) was used when incubation in blocking lysates at 4 °C for 3 h to avoid the non-specific RNA–protein binding. Then washed twice with ice-cold lysis buffer, three times with the low salt buffer (0.1% SDS, 1% Triton X-100, 2 mM EDTA, 20 mM Tris–HCl, pH 8.0, and 150 mM NaCl), and once with the high salt buffer (0.1% SDS, 1% Triton X-100, 2 mM EDTA, 20 mM Tris–HCl, pH 8.0, and 500 mM NaCl). Finally, the bound DNAs or RNAs were extracted and purified for qPCR.

### Nascent-transcribed RNAs detection

This assay was performed with the click-iT^®^ Nascent RNA Capture Kit (ThermoFisher) according to the manufacturer’s instructions. Briefly, cells (5 × 10^5^/well) were seeded in six-well plate and labeled by adding EU to cell culture medium (0.5 mM for 2 h), followed by cell collection for RNA extraction using TRIzol regent. Then 5 μg EU-RNAs were prepared to click to Biotin-Azide (0.5 mM) in Click-iT reaction cocktail for 30 min, and the Biotin-EU RNAs were purified. Afterwards, about 500 ng quantified RNAs were yielded to incubate with 50 μL Streptavidin T1 magnetic beads and washed five times, finally reverse transcriptase-mediated cDNA synthesis on beads should be immediately performed to be further used for qRT-PCR to detect nascent-transcribed RNAs of interested.

### Statistical analysis

All experiments were performed at least in triplicate and used the Student’s *t*-tests to analyze statistical difference only if specified. Differences were regarded as significant when *p* < 0.05.

## Result

### ARHGAP5-AS1 was upregulated to promote chemoresistance

**Fig. 1:**
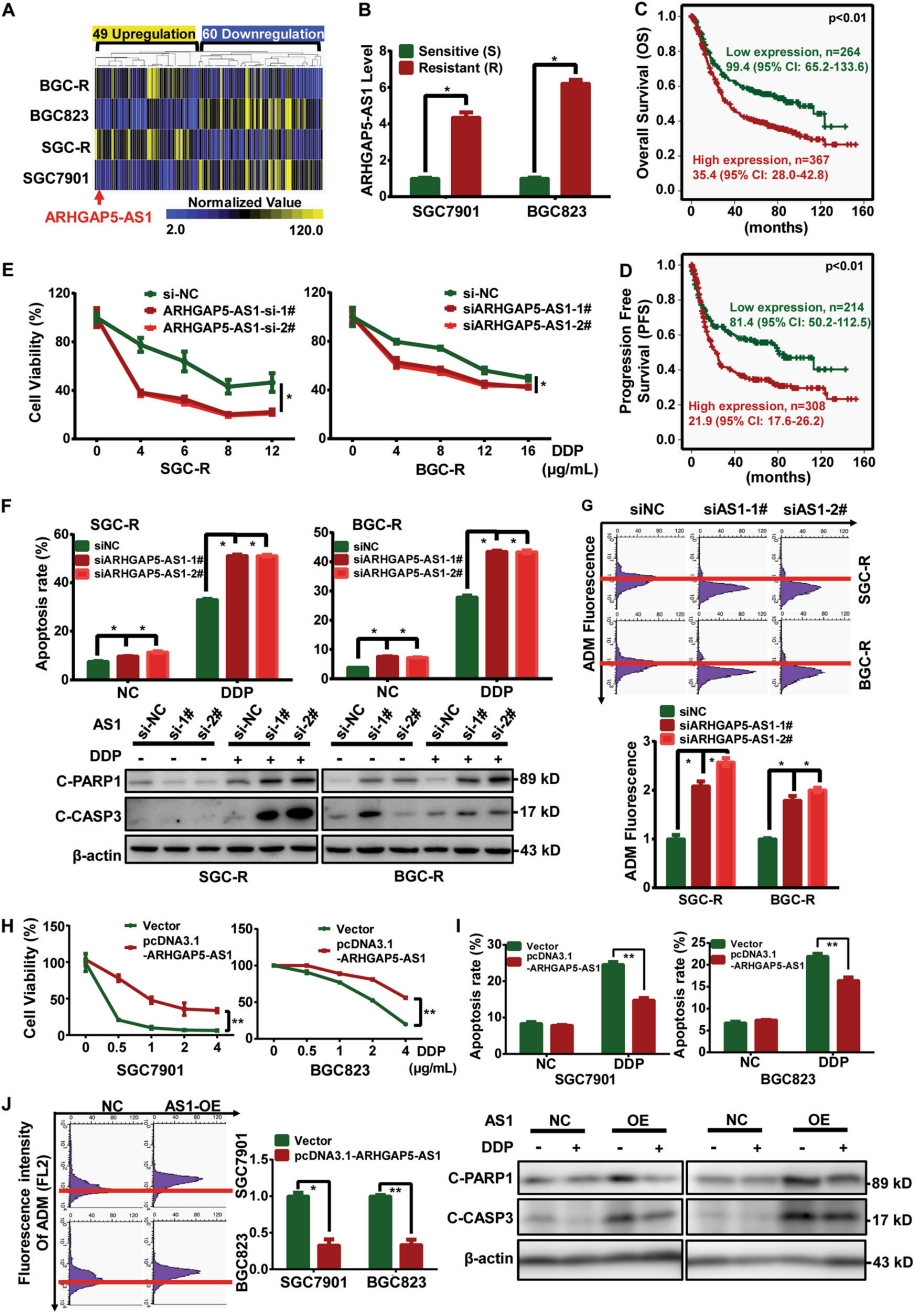
ARHGAP5-AS1 was upregulated to promote chemoresistance in cancer cells. **a** Heatmap visualization of differential lncRNAs expression (49 upregulated and 60 downregulated) in chemosensitive or resistant cells. **b** The expression of ARHGAP5-AS1 in chemosensitive or resistant SGC7901 and BGC823 cells were detected by qRT-PCR. Data were presented as the mean ± SD, *n* = 3. *p < 0.05 (Student’s *t*-test). The Kaplan–Meier curve analysis on the impact of ARHGAP5-AS1 expression on overall survival (**c**) and progression‑free survival (**d**). *p*-Value was calculated by Log Rank test. **e** The effect of ARHGAP5-AS1 knockdown on viability of resistant cells with or without DDP treatment for 36 h were detected using MTS assay. Experiments were all repeated three times and the representative data were shown. The asterisks indicate the statistical significance (*p* < 0.05). **f** Resistant cells transfected with control siRNA (siNC) or ARHGAP5-AS1 siRNAs were treated with or without DDP (5 μg/mL) for 36 h and the apoptosis was measured using flow cytometry (top panel) as well as Western Blotting (bottom panel). **g** ARHGAP5-AS1 was knocked down in resistant cells and the intracellular concentration of doxorubicin hydrochloride (ADM) in ADM (5 μg/mL, 2 h)-incubated resistant cells before and after ARHGAP5-AS1 knockdown were assessed using flow cytometry (the raw fluorescence intensity measured using FL2 channel was shown as top panel and the normalized value was well statistical and shown as bottom panel). **h** The empty pcDNA3.1 vector or pcDNA3.1-ARHGAP5-AS1 was transfected into sensitive cells and relative cell viability before or after DDP treatment for 24 h were detected using MTS assay. **i** The apoptosis of sensitive cells with or without ARHGAP5-AS1 overexpression in the presence or absence of DDP treatment (2 μg/mL, 24 h) were measured using flow cytometry (top panel) as well as Western Blotting (bottom panel). **j** The intracellular concentration of ADM in sensitive cells before and after ARHGAP5-AS1 overexpression were determined as in **g**

To clarify the relevance of deregulated lncRNAs to chemoresistance, we profiled lncRNAs expression in two paired drug-sensitive/resistant cells (SGC7901/SGC-R and BGC823/BGC-R) as previously reported^25,26^. In total, 109 differentially expressed lncRNAs were found, of which 49 were upregulated and 60 were downregulated in chemo-resistant cells. ARHGAP5-AS1 was one of the most eminent transcripts significantly upregulated in resistant cells (Fig. 1a). We firstly validated the screening results by qRT-PCR, showing ARHGAP5-AS1 was dominantly overexpressed in resistant cells (Fig. 1b). Besides, clinical data from the Cancer Genome Atlas (TCGA) database showed that higher ARHGAP5-AS1 expression level was correlated to shorter overall survival (Fig. 1c) and progression-free survival (Fig. 1d) in gastric cancer patients (Table 1). Hence, we assumed that the high level of ARHGAP5-AS1 contributed to chemoresistance in gastric cancer. In fact, downregulation of ARHGAP5-AS1 (Supplementary Fig. 1a) in resistant cells evidently reversed the resistance to chemotherapeutic drugs including cisplatin (DDP), ADM, and 5-FU (Fig. 1e and Supplementary Fig. 1b), enhanced drug-induced apoptosis (Fig. 1f and Supplementary Fig. 1c–e) and increased the intracellular drug concentration (Fig. 1g). On the contrary, overexpression of ARHGAP5-AS1 in sensitive cells (Supplementary Fig. 1f) dramatically attenuated drug-induced viability inhibition (Fig. 1h and Supplementary Fig. 1g), reduced drug-activated apoptosis (Fig. 1i and Supplementary Fig. 1h–j), and decreased intracellular drug concentration (Fig. 1j). Taken together, these results indicated that ARHGAP5-AS1 plays a critical role in promoting chemoresistance.

**Table 1:** Cox regression analysis of overall survival and progression free survival in gastric cancer

Variable	Overall survival				Progression-free survival			
	Univariate		Multivariate		Univariate		Multivariate	
	RR (95% CI)	*p*-value	RR (95% CI)	*p*-value	RR (95% CI)	*p*-value	RR (95% CI)	*p*-value
*Gender*								
Female	1	**0.026**	1	0.724	1	**<0.05**	1	0.897
Male	1.3 (1.0–1.6)		1.1 (0.8–1.4)		0.8 (0.6–1.0)		1.0 (0.8–1.3)	
*Lauren type*								
Intestinal	1		1		1		1	
Diffuse	1.2 (0.9–1.5)	0.158	1.2 (0.9–1.5)	0.264	1.2 (0.9–1.6)	0.077	1.1 (0.8–1.4)	0.661
Mixed	1.0 (0.6–1.6)	0.863	1.0 (0.6–1.9)	0.882	1.4 (0.9–2.4)	0.157	1.2 (0.7–2.1)	0.412
*TNM stage*								
I	1		1		1		1	
II	1.5 (0.8–2.7)	0.219	1.7 (0.8–3.4)	0.144	1.6 (0.8–2.9)	0.15	1.5 (0.8–2.9)	0.182
III	4.3 (2.5–7.3)	**<0.01**	4.1 (2.1–7.9)	**<0.01**	4.2 (2.4–7.4)	**<0.01**	3.9 (2.2–7.1)	**<0.01**
IV	7.1 (4.0–12.4)	**<0.01**	8.9 (4.6–17.2)	**<0.01**	9.0 (5.0–16.1)	**<0.01**	8.5 (4.7–15.2)	**<0.01**
*ARHGAP5-AS1*								
Low expression	1	**<0.01**	1	**<0.01**	1	**<0.01**	1	**<0.01**
High expression	1.6 (1.2–2.0)		1.5 (1.2–2.0)		1.6 (1.2–2.1)		1.4 (1.1–1.8)	

The bold values indicates the differences are remarkably significant (*p* < 0.05)

### Autophagy-dependent degradation of ARHGAP5-AS1

**Fig. 2:**
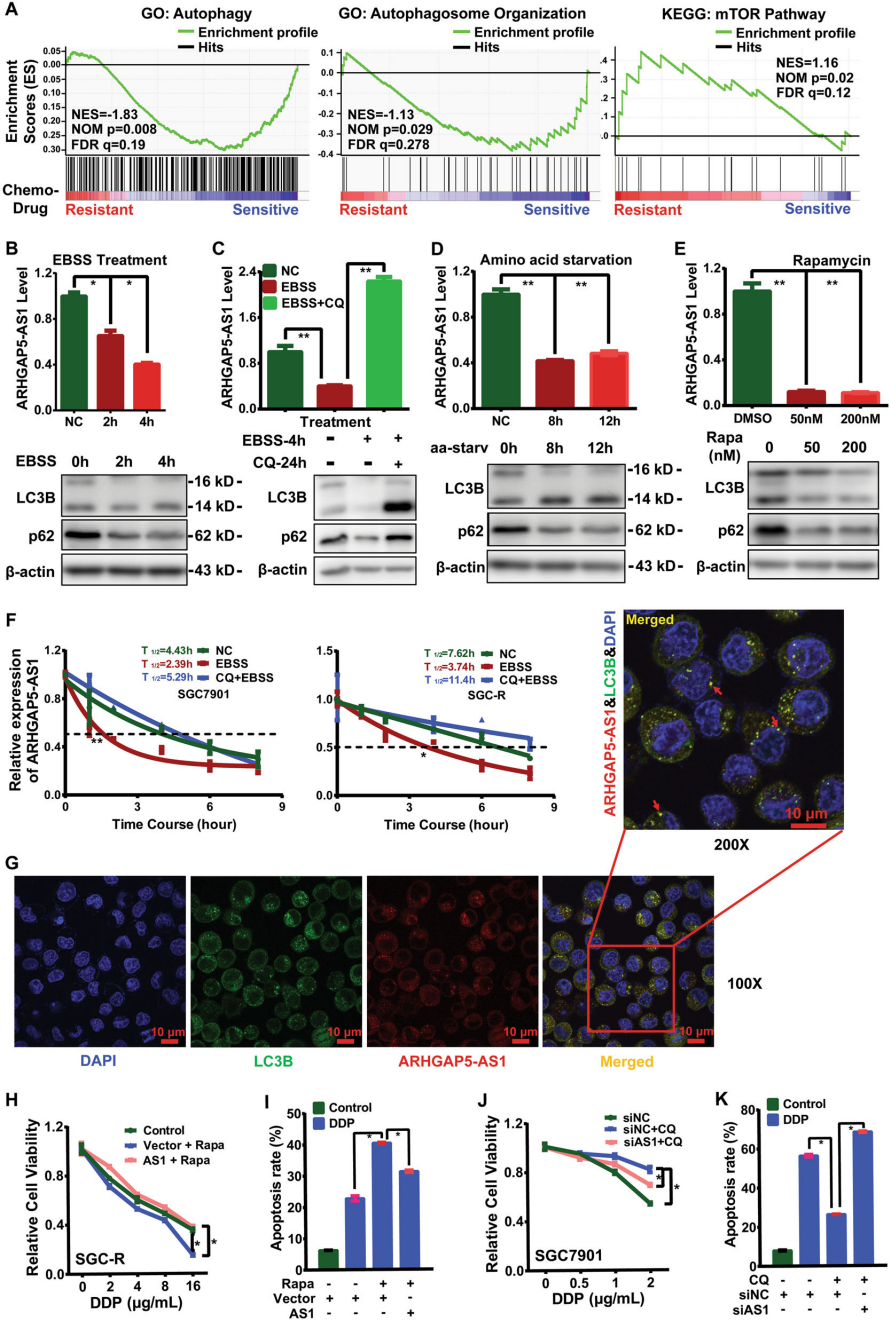
Autophagy-dependent degradation of ARHGAP5-AS1. **a** Gene set enrichment analysis (GSEA) of deregulated transcripts in chemoresistant or sensitive cells. Red indicates drug resistant; blue indicates drug sensitive. **b** The effect of autophagy induction using EBSS treatment for 2 or 4 h on ARHGAP5-AS1 level in SGC-R were determined by qRT-PCR (top panel). Autophagy-related markers were detected by Western Blotting (bottom panel). **c** The effect of CQ (50 μM, 24 h) combined EBSS (4 h) treatment on ARHGAP5-AS1 expression in SGC-R cells were determined by qRT-PCR (top panel). Data were presented as the mean ± SD, *n* = 3. **p < 0.01 (Student’s *t*-test). The dynamic autophagic effect was assessed using Western Blotting (bottom panel). **d** ARHGAP5-AS1 expression in SGC-R cells with amino acid starvation for 8 or 12 h were detected by qRT-PCR (top panel). Data were presented as the mean ± SD, *n* = 3. **p < 0.01 (Student’s *t*-test). Markers for autophagy were measured using Western Blotting (bottom panel). **e** The effect of rapamycin (48 h) on ARHGAP5-AS1 expression in SGC-R were analyzed by qRT-PCR (top panel). The induction of autophagy was assessed by Western Blotting (bottom panel). **f** The half-life of ARHGAP5-AS1 in various SGC-R cells treated as indicated were determined by RT-PCR. Half-life curves were plotted on nonlinear fitting and regression (one phase decay curve fit). **g** The colocalization of ARHGAP5-AS with LC3B in SGC-R cells treated with CQ (50 μM, 12 h) and EBSS (2 h) was detected by FISH and IFC assay under confocal microscope (original magnification, ×100). Scale bar: 10 μm. **h** and **i** The viability (**h**) and apoptosis (**i**) of SGC-R cells treated as indicated were detected using MTS or flow cytometry assay, respectively. Experiments were all repeated three times and the representative data were shown. The asterisks indicate the statistical significance (*p* < 0.05). **j** and **k** The viability (**j**) and apoptosis (**k**) of SGC7901 cells treated as indicated were detected using MTS or flow cytometry assay, respectively. Experiments were all repeated three times and the representative data were shown. The asterisks indicate the statistical significance (*p* < 0.05)

In consistence with inhibition of autophagy in chemo-resistant cancer cells^25^, gene set enrichment analysis (GSEA) based on expression profiling indicated that autophagy was impaired and mTOR was activated in chemo-resistant cancer cells (Fig. 2a–c). Since autophagy was important for the homeostasis of proteins even organelles, we wonder whether autophagy inactivation was also relevant to upregulation of ARHGAP5-AS1 in chemo-resistant cells. Indeed, the expression of ARHGAP5-AS1 was notably dropped once autophagy was activated by EBSS treatment (Fig. 2b and Supplementary Fig. 2a). However, the level of ARHGAP5-AS1 was remarkably recovered upon the addition of CQ, an autophagy-lysosomal inhibitor (Fig. 2c and Supplementary Fig. 2b). Similarly, ARHGAP5-AS1 expression was decreased when autophagy was induced by amino acid starvation (Fig. 2d and Supplementary Fig. 2c) or rapamycin treatment (Fig. 2e and Supplementary Fig. 2d). Furthermore, autophagy activation could significantly attenuate the stability of ARHGAP5-AS1, with its half-life shortening from 4.43 and 7.62 to 2.39 and 3.74 h in chemo-sensitive and resistant cells, which was reversed by CQ (Fig. 2f and Supplementary Fig. 2e). Intriguingly, ARHGAP5-AS1 was able to co-localize with LC3B-positive puncta (Fig. 2g). Overall, these data strongly implicate that ARHGAP5-AS1 might be degraded by autophagy.

As we recently reported^25^, autophagy inactivation contributed to chemoresistance in gastric cancer. Thus, we wonder whether autophagy-dependent regulation of ARHGAP5-AS1 expression was relevant to chemoresistance. Indeed, autophagy activation by mTOR inhibitor rapamycin in chemoresistant gastric cancer cells restored drug sensitivity, which was impaired by overexpressing exogenous ARHGAP5-AS1 (Fig. 2h, i). In contrast, inhibition of autophagy by CQ conferred drug resistance in chemosensitive gastric cancer cells only in the absence of ARHGAP5-AS1 knock-down (Fig. 2j, k). Taken together, autophagic degradation of ARHGAP5-AS1 was relevant to chemosensitivity.

### SQSTM1 recruited ARHGAP5-AS1 for autophagic degradation

**Fig. 3:**
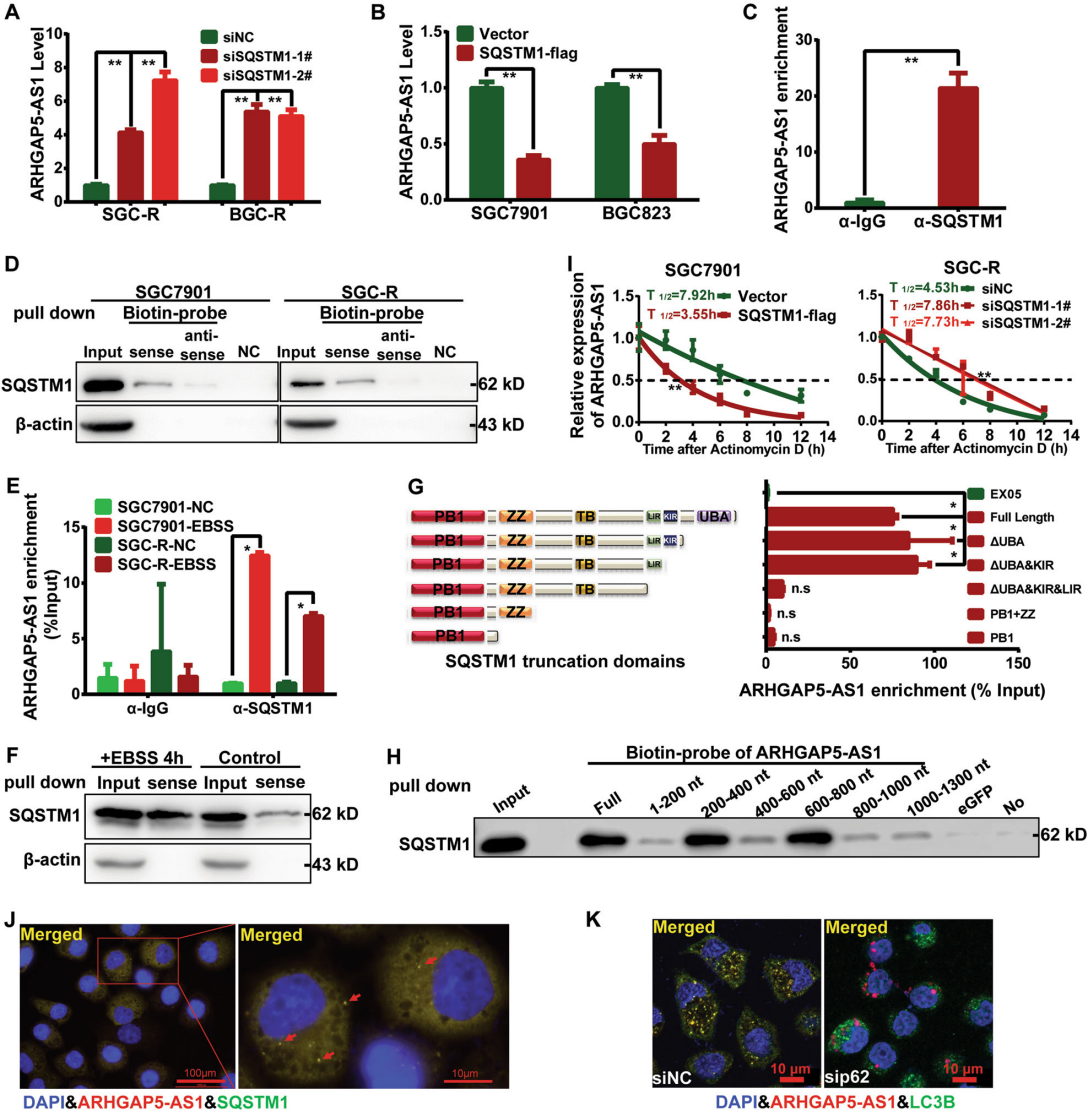
SQSTM1 recruited ARHGAP5-AS1 for autophagic degradation. **a** ARHGAP5-AS1 expression in resistant cells after transfecting siNC or SQSTM1 siRNAs was assessed using qRT-PCR. **p < 0.01. **b** ARHGAP5-AS1 expression in sensitive cells after SQSTM1 overexpression was detected by qRT-PCR. **c** The interaction of ARHGAP5-AS1 with SQSTM1 in SGC-R cells was analyzed by RIP assay followed with qRT-PCR. IgG was served as the negative control. **d** RNA pull down assay was performed to verify the binding of SQSTM1 to ARHGAP5-AS1 in SGC7901 or SGC-R cells. β-actin was served as negative control. NC: no probe. The interaction of ARHGAP5-AS1 with SQSTM1 in SGC-R cells before and after EBSS treatment (4 h) were analyzed using RIP assay (**e**) and RNA pull down assay (**f**). **g** The interaction of various SQSTM1 constructs with ARHGAP5-AS1 were analyzed using RIP assay. **h** The interaction of various ARHGAP5-AS1 fragments with SQSTM1 was analyzed by RNA pull down assay. **i** Half-life of ARHGAP5-AS1 in SGC7901 and SGC-R cells with different SQSTM1 expression status were determined by qRT-PCR. The colocalization of ARHGAP5-AS with SQSTM1 (**j**) and LC3B (**k**) in SGC-R cells treated as indicated were analyzed by combined FISH and IFC assay (original magnification, ×100). Scale bar: 100 μm (**j**) and 10 μm (**k**)

To further investigate the mechanism underlying the autophagy-dependent degradation of ARHGAP5-AS1, we focused on SQSTM1, which played an essential role in transporting the ready-to-degradation proteins to autophagosome, was upregulated in resistant cells^25^. Knockdown of SQSTM1 in resistant cells elevated ARHGAP5-AS1 expression (Fig. 3a and Supplementary Fig. 3a), whereas the overexpression of SQSTM1 in sensitive cells reduced ARHGAP5-AS1 level (Fig. 3b and Supplementary Fig. 3b). Both RIP and RNA pull down assay demonstrated a direct interaction between SQSTM1 and ARHGAP5-AS1 indeed (Fig. 3c, d), which was markedly enhanced after autophagy activation (Fig. 3e, f). In addition, ARHGAP5-AS1 was co-localized with SQSTM1 in vivo (Fig. 3g and Supplementary Fig. 3c). To further characterize the interaction of ARHGAP5-AS1 with SQSTM1, we constructed different truncation segments of SQSTM1 and ARHGAP5-AS1. Finally, we found that the LC3-interacting region (LIR) domain of SQSTM1 was mandatory for binding to ARHGAP5-AS1 (Fig. 3h and Supplementary Fig. 3d). Correspondingly, SQSTM1 showed an inclination to bind the region between 200 nt and 800 nt in ARHGAP5-AS1 (Fig. 3i). Meanwhile, in line with our predication, knockdown or overexpression of SQSTM1 could dramatically lengthen or shorten the half-life of ARHGAP5-AS1, respectively (Fig. 3j and Supplementary Fig. 3e). Moreover, the colocalization of ARHGAP5-AS1 with LC3B-posotive puncta was reduced once SQSTM1 was knocked down (Fig. 3k and Supplementary Fig. 3f). In conclusion, SQSTM1 recruits ARHGAP5-AS1 for autophagic degradation.

### ARHGAP5-AS1-stimulated ARHGAP5 transcription

**Fig. 4:**
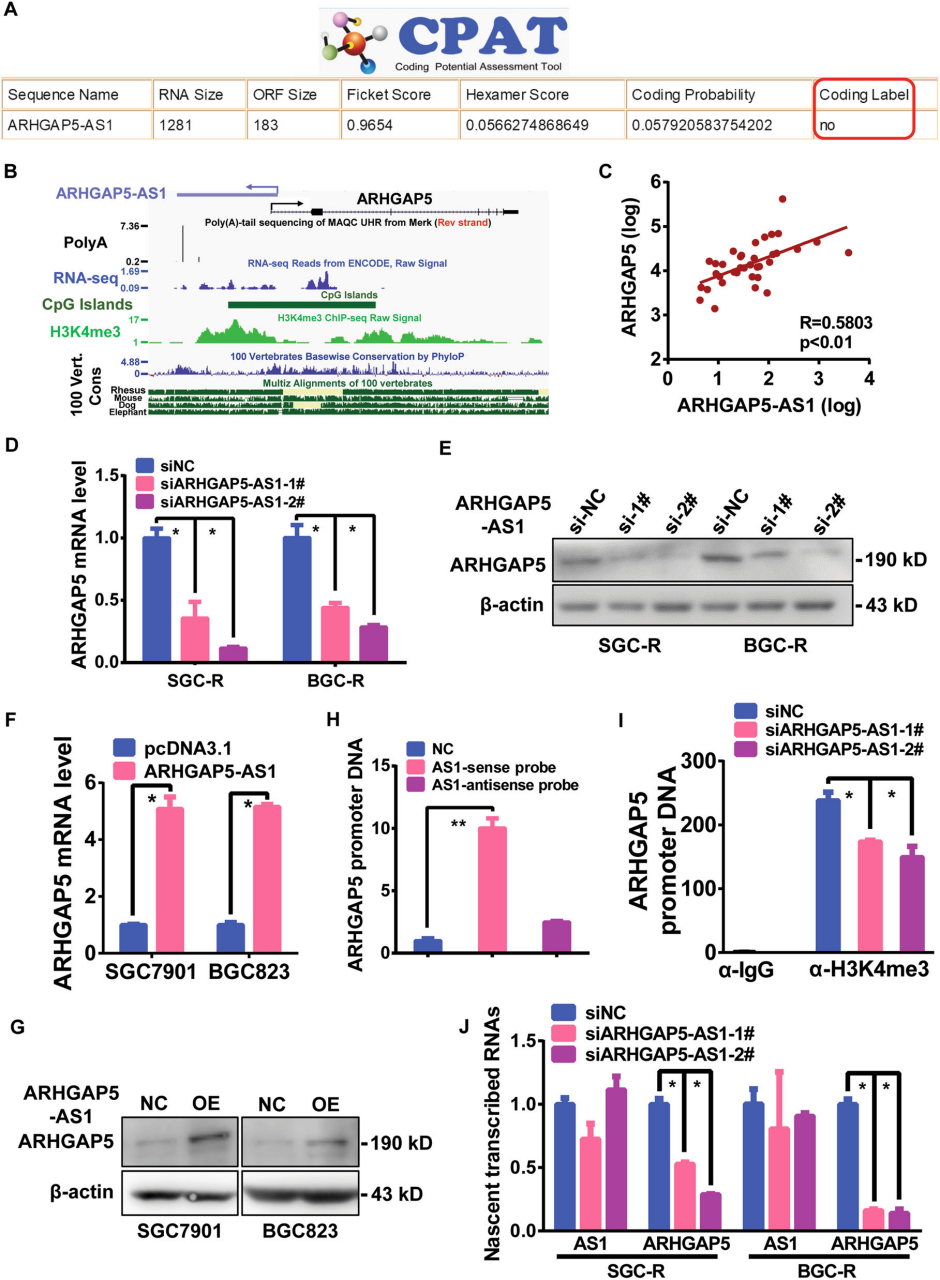
ARHGAP5-AS1 stimulated ARHGAP5 transcription. **a** The coding potential of ARHGAP5-AS1 was predicted by CPAT. **b** The genomic loci of ARHGAP5-AS1. RNA-seq raw signal showed the primary structure of ARHGAP5-AS1. Chromatin marks of transcription initiation (histoneH3 lysine 4 trimethylation, H3K4me3) and CpG Islands defined the beginning of ARHGAP5-AS1, and sequencing of poly-adenylation ends (3′ Poly (A)-seq) defined the precise ends of this transcript. The 100 Vert. Cons scores described conservation of ARHGAP5-AS1 in mammals. All of these raw data were analyzed using UCSC genome browser. **c** Correlation analysis of ARHGAP5-AS1 with ARHGAP5 mRNA. The expression data was derived from TCGA. *R* meaned Pearson correlation coefficient and *p* represented significance. Expression of ARHGAP5 in SGC-R and BGC-R cells transfected with ARHGAP5-AS1 siRNAs were analyzed by qRT-PCR (**d**) and Western blotting (**e**). Expression of ARHGAP5 in SGC7901 and BGC823 cells with ARHGAP5-AS1 overexpression were measured by qRT-PCR (**f**) and Western blotting (**g**). **h** Sense or antisense probe of ARHGAP5-AS1 were biotin labeled and used to pull down ARHGAP5 promoter DNA. **i** ChIP was performed to assess the differential status of H3K4me3 around ARHGAP5 promoter in SGC-R cells with ARHGAP5-AS1 knockdown. **j** The nascent transcribed ARHGAP5 mRNA in SGC-R cells after knocking down ARHGAP5-AS1 was analyzed by Click it assay. *p < 0.05 (Student’s *t-*test)

An analysis of the protein-coding potential of ARHGAP5-AS1 validated it as a noncoding RNA (Fig. 4a). Based on genomic loci and annotation in the UCSC browser, ARHGAP5-AS1 was arranged in antisense orientation with respect to its protein coding partner, ARHGAP5 (Fig. 4b). Besides, expression data from TCGA revealed a positive correlation between ARHGAP5-AS1 and ARHGAP5 mRNA (Fig. 4c), suggesting that ARHGAP5-AS1 could potentially function as a regulator of ARHGAP5 expression. Knocking down ARHGAP5-AS1 in resistant cells resulted in the reduction of ARHGAP5 mRNA and protein abundance (Fig. 4d, e and Supplementary Fig. 4a). In contrast, ARHGAP5-AS1 overexpression increased the abundance of ARHGAP5 mRNA and protein in sensitive cells (Fig. 4f, g and Supplementary Fig. 4b). Given the functional mode of natural antisense transcript (NAT) and the fact that ARHGAP5-AS1 was head-to-head transcript as for ARHGAP5 mRNA, we speculated that ARHGAP5-AS1 could affect the transcription of ARHGAP5. In fact, ARHGAP5-AS1 was able to interact with ARHGAP5 (Fig. 4h). ARHGAP5-AS1 depletion resulted in the loss of H3K4me3 modification at the ARHGAP5 core promoter area (Fig. 4i and Supplementary Fig. 4c), accompanied by reduced generation of nascent ARHGAP5 mRNA (Fig. 4j). On the contrary, the increasing ARHGAP5-AS1 level could promote transcription of ARHGAP5 mRNA (Supplementary Fig. 4d). Collectively, ARHGAP5-AS1 interacts with ARHGAP5 promoter to promote its transcription.

### ARHGAP5-AS1 stabilized ARHGAP5 mRNA in the cytoplasm

**Fig. 5:**
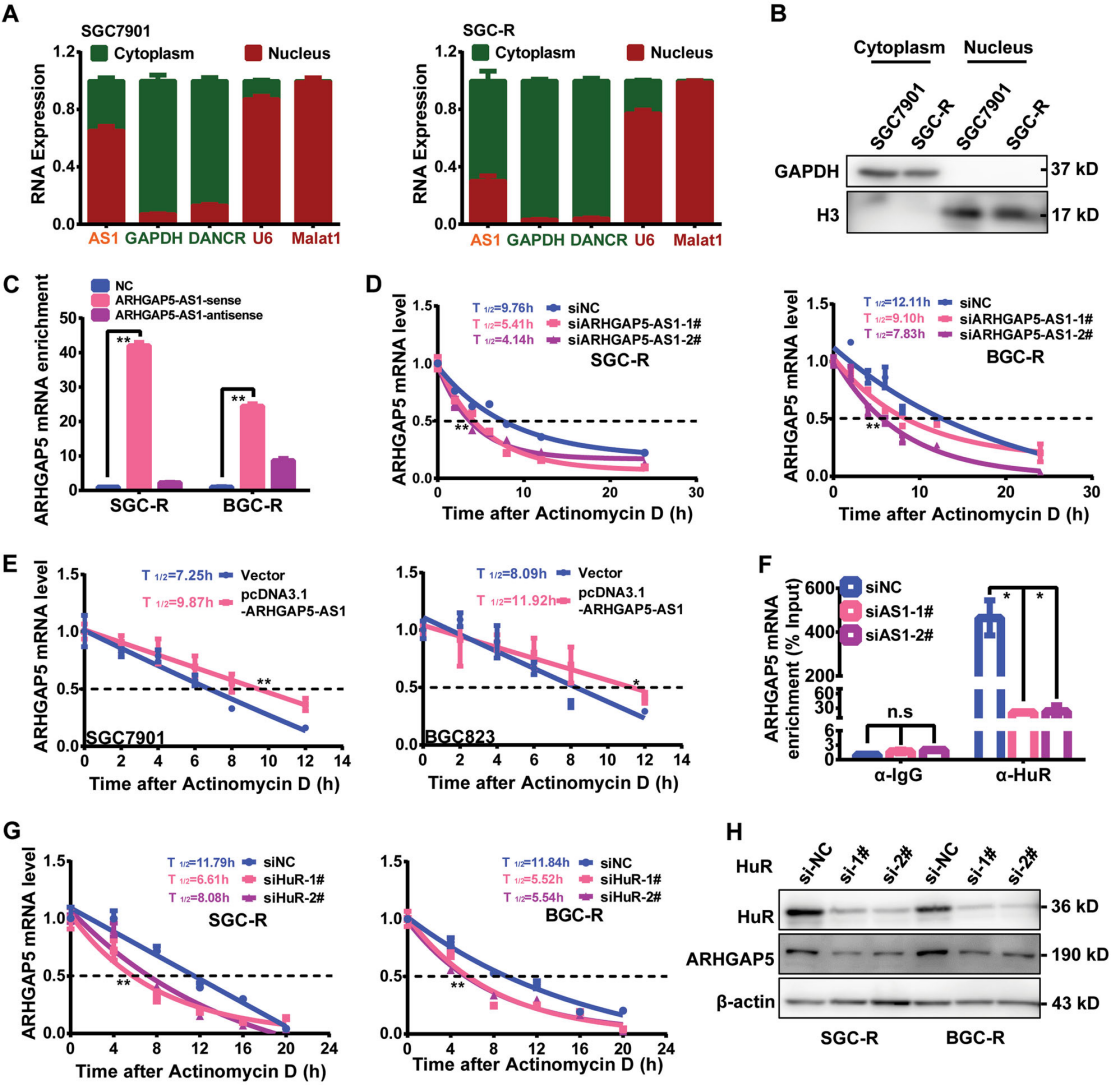
ARHGAP5-AS1 stabilized ARHGAP5 mRNA in the cytoplasm. **a** The subcellular localization of ARHGAP5-AS1 in SGC7901/SGC-R cells were determined by qRT-PCR after extracting RNAs from cytoplasmic and nuclear extracts. GAPDH/DANCR and U6/Malat1 were used as the cytoplasmic and nuclear RNA controls, respectively. **b** The separation of cytoplasmic and nuclear fractions was validated by Western Blotting. GAPDH and H3 were served as cytoplasmic and nuclear protein controls, respectively. **c** Biotin pull down assay was used to verify the interaction of ARHGAP5-AS1 with ARHGAP5 mRNA. NC meaned no probe; both sense and antisense probe were biotin labeled. The enrichment of ARHGAP5 mRNA was measured by qRT-PCR. The change of ARHGAP5 half-life after knocking down ARHGAP5-AS1 levels in SGC-R and BGC-R cells (**d**) or overexpressing ARHGAP5-AS1 in SGC7901 and BGC823 cells (**e**) were analyzed as in Fig. 2f. **f** The effect of ARHGAP5-AS1 downregulation on the interaction of ARHGAP5 mRNA with HuR protein was analyzed by RIP assay. **g** Change of ARHGAP5 half-life after knocking down HuR levels in SGC-R and BGC-R cells. **h** ARHGAP5 protein expression after knocking down HuR was determined by Western Blotting

The interaction of SQSTM1 with ARHGAP5-AS1 indicated that ARHGAP5-AS1 was also located in the cytoplasm (Figs. 2g and 3j). Indeed, ARHGAP5-AS1 was distributed in both cytoplasmic and nuclear fractions (Fig. 5a, b). We wondered whether ARHGAP5-AS1 has any functional relevance in the cytoplasm. Interestingly, we found an interaction between ARHGAP5-AS1 and ARHGAP5 mRNA using biotin pull down assay (Fig. 5c and Supplementary Fig. 5a). Once ARHGAP5-AS1 was knocked down, the half-life of ARHGAP5 mRNA was substantially decreased (Fig. 5d), whereas overexpressed ARHGAP5-AS1 could dramatically elongate the half-life of ARHGAP5 mRNA (Fig. 5e). As the RNA-binding protein HuR was important to regulate the stability of mRNA^27,28^, we explored if ARHGAP5-AS1 can regulate the stability of ARHGAP5 mRNA through affecting its binding to HuR. As a result, we found that ARHGAP5 mRNA could potentially interact with HuR through bioinformatic prediction (Supplementary Fig. 5b, c). RNA immunoprecipitation indeed confirmed the interaction of HuR with ARHGAP5 mRNA, which was abrogated by knocking down ARHGAP5-AS1 (Fig. 5f). Moreover, downregulation of HuR dramatically shorten the half-life of ARHGAP5 mRNA and reduced ARHGAP5 protein expression in resistant cells (Fig. 5g, h). To sum up, ARHGAP5-AS1 could bind and stabilize ARHGAP5 mRNA through enhancing its interaction with HuR.

### ARHGAP5-AS1 recruited METTL3 for m^6^A modification of ARHGAP5 mRNA

**Fig. 6:**
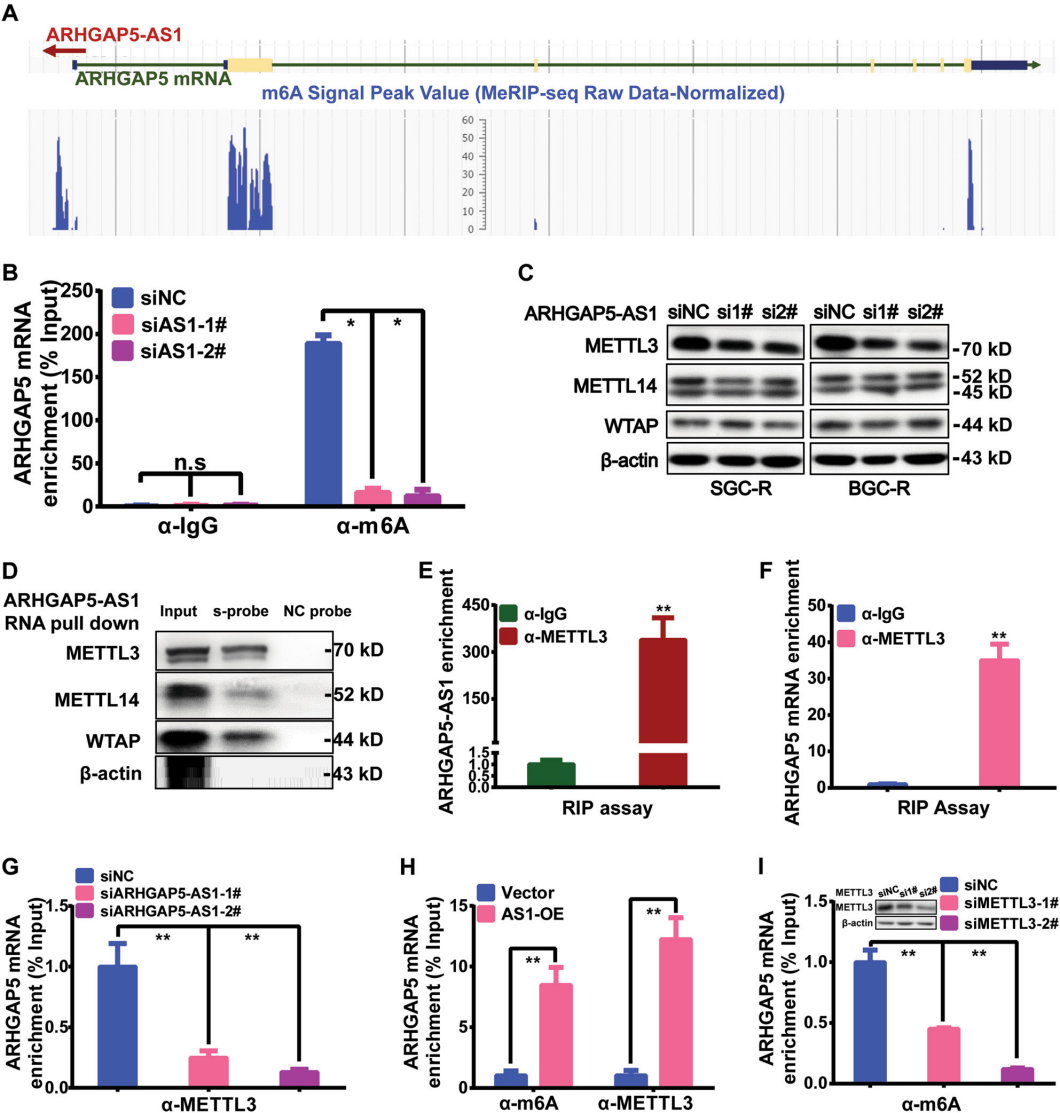
ARHGAP5-AS1 recruited METTL3 for m^6^A modification of ARHGAP5 mRNA. **a** RMBase v2.0 was used to screen m^6^A modification sites of ARHGAP5 mRNA. Raw signal value for peak calling was derived from MeRIP-seq data. The scale ruler indicated normalized peak value. **b** The effect of ARHGAP5-AS1 downregulation on m^6^A modification of ARHGAP5 mRNA was assessed by RIP assay. **c** M^6^A methyltransferase levels in resistant cells after knocking down expression of ARHGAP5-AS1 were detected using Western Blotting. **d** RNA pull down assay using ARHGAP5-AS1 biotin-labeled probes was performed to analyze the interaction between ARHGAP5-AS1 and m^6^A methyltransferase. **e**, **f** The binding capacity of ARHGAP5-AS1 (**e**) or ARHGAP5 mRNA (**f**) with METTL3 were determined by RIP assay. **g** Interaction of ARHGAP5 mRNA with METTL3 after ARHGAP5-AS1 downregulation was assessed by RIP assay. **h** Effect of ARHGAP5-AS1 overexpression on m^6^A modification and METTL3 interaction of ARHGAP5 were detected by RIP. **i** The effect of METTL3 downregulation on m^6^A modification of ARHGAP5 mRNA was assessed by RIP assay

Recently, m^6^A modification was found to influence mRNA stability through RNA-binding proteins such as HuR^29,30^. Therefore, we explored the relevance of m^6^A modification to the regulation of ARHGAP5 mRNA stability. Both RMBase and SRAMP analysis indicated potential m^6^A modification of ARHGAP5 mRNA (Fig. 6a and Supplementary Fig. 6a). Anti-m^6^A antibody indeed enriched a significant amount of ARHGAP5 mRNA (Fig. 6b). ARHGAP5 mRNA was also able to interact with the core component of m^6^A methyltransferases METTL3 (Fig. 6c). When METTL3 was depleted, m^6^A modification of ARHGAP5 was significantly reduced (Fig. 6d and Supplementary Fig. 6b, c). Interestingly, ARHGAP5-AS1 was also able to interact with METTL3 and other components of m^6^A methyltransferase, such as METTL14 and WTAP (Fig. 6e, f). Once it was depleted, m^6^A modification of ARHGAP5 was reduced (Fig. 6g), accompanied by reduced interaction of METTL3 with ARHGAP5 mRNA (Fig. 6h). On the contrary, m^6^A modification and METTL3 binding of ARHGAP5 mRNA were both elevated when ARHGAP5-AS1 was overexpressed (Fig. 6i). In summary, ARHGAP5-AS1 could effectively recruit METTL3 to facilitate m^6^A modification of ARHGAP5 mRNA.

### ARHGAP5 promoted chemoresistance in gastric cancer

**Fig. 7:**
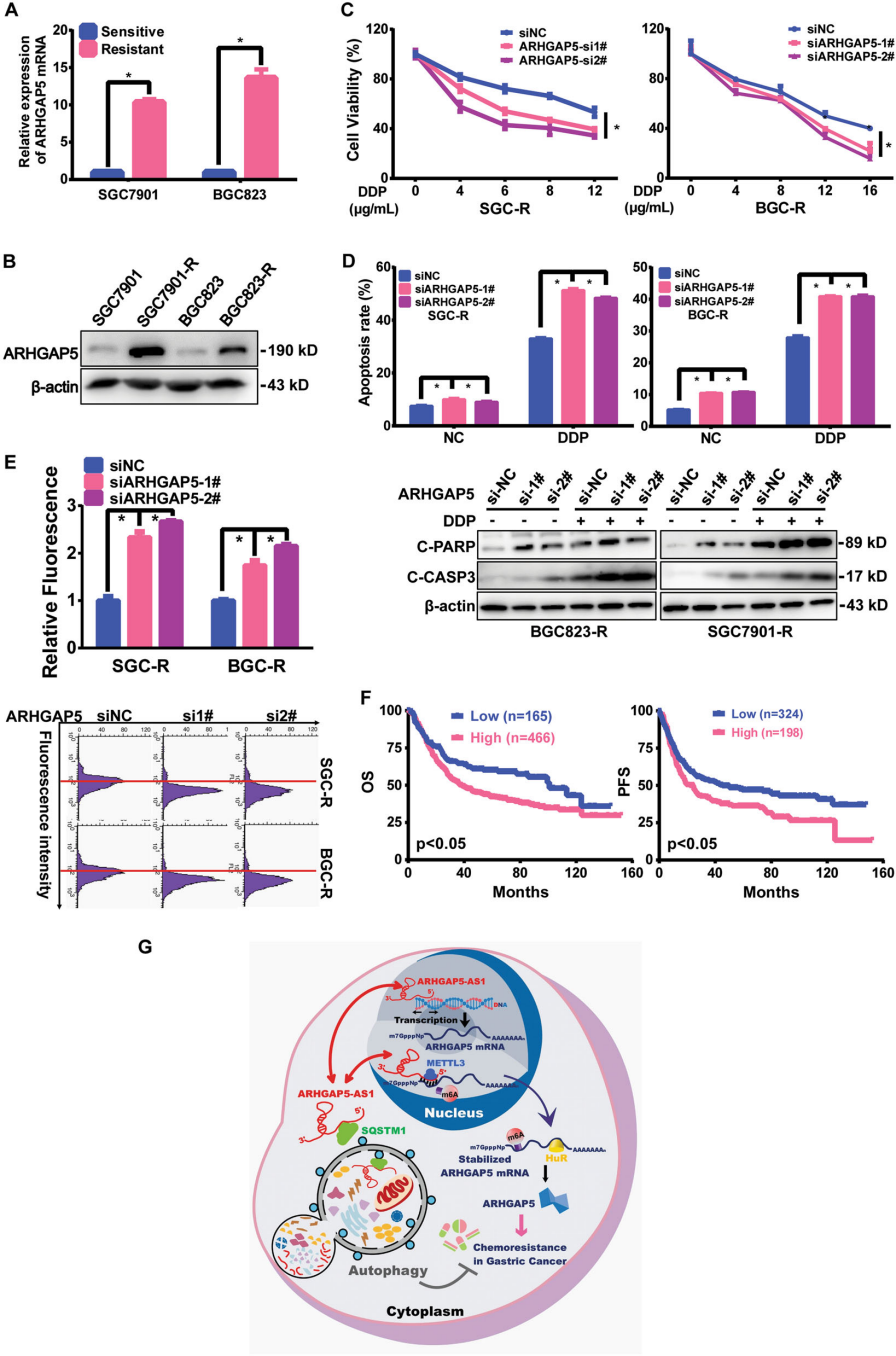
ARHGAP5 promoted chemoresistance in gastric cancer. The expression of ARHGAP5 mRNA (**a**) and protein (**b**) in chemosensitive or resistant cells were detected by qRT-PCR and Western Blotting, respectively. **c** The effect of ARHGAP5 knockdown on viability of resistant cells with or without DDP treatment for 36 h were detected using MTS assay. **d** siNC or ARHGAP5 siRNAs were transfected to resistant cells and apoptosis after DDP (5 μg/mL) treatment for 36 h was measured using flow cytometry (top panel) as well as Western Blotting (bottom panel). **e** ARHGAP5 was knocked down in resistant cells and the intracellular concentration of ADM was assessed using flow cytometry (top panel: the raw fluorescence intensity; bottom panel: the normalized fluorescence value). **f** The Kaplan–Meier curve analysis on the impact of ARHGAP5 expression on overall survival (left) and progression‑free survival (right) of gastric cancer patients. *p*-Value was calculated by Log Rank test. **g** A schematic illustration of the proposed model depicting the role of ARHGAP5-AS1, the autophagic-regulated lncRNA, in promoting chemoresistance of gastric cancer. It can not only stimulate the transcription of ARHGAP5 in the nucleus but also recruit METTL3 for the m^6^A modification and subsequent stabilization of ARHGAP5 mRNA in the cytoplasm

All of these results indicated that ARHGAP5 was stimulated by ARHGAP5-AS1 and may contribute to chemoresistance. In fact, the expression of ARHGAP5 was elevated in resistant cells (Fig. 7a, b). Downregulation of ARHGAP5 evidently reversed chemoresistance (Fig. 7c and Supplementary Fig. 7a, b), increased drug-induced apoptosis (Fig. 7d and Supplementary Fig. 7c, d) and intracellular drug concentration (Fig. 7e). In addition, high expression of ARHGAP5 was associated with shorter overall survival and progression-free survival (Fig. 7f). Importantly, chemoresistance induced by ARHGAP5-AS1 overexpression was remarkably reversed by ARHGAP5 depletion (Supplementary Fig. 7e–h). Therefore, ARHGAP5-AS1 stimulates ARHGAP5 expression so as to promote chemoresistance.

## Discussion

In this study, we reported a NAT lncRNA promoting chemoresistance in gastric cancer. Increasing evidence confirmed the involvement of deregulated lncRNAs in the pathogenesis of various diseases including cancers. Like protein-coding mRNAs, lncRNAs were regulated by the balance between biogenesis and degradation. While the biogenesis of lncRNAs including gene transcription and post-transcriptional processes, such as alternative splicing was believed to resemble mRNA biogenesis, the degradation of lncRNAs was not distinct from mRNA degradation. Generally, the degradation of mRNAs occurs in the cytoplasm mainly by Xrn1-mediated decapping and 5-to-3′exonuclease digestion. In contrast, some lncRNAs can be degraded by exosome or Nrd1/Nab3/Sen1 complex in the nucleus or nonsense-mediated decay (NMD) in the cytoplasm^16^. However, accumulated findings indicated that lncRNAs undergoing NMD is only 4.47–14.11% variable in different species^31^. Moreover, exosome mainly involved in the degradation of ribosomal RNAs, sn/snoRNAs, and hypomodified tRNAs through Rrp6p and Dis3p exonucleases^32,33^. Therefore, there must exist other pathways for the degradation of LncRNAs.

Interestingly, the autophagy-dependent RNA catabolism has been discovered in yeast and RNAs are broken down by Pnp1/Urh1 through Rny1-mediated transportation to the vacuole^34,35^. Since autophagy was traditionally recognized to degrade protein, the degradation of RNAs in autolysosomes was specifically named as RNautophagy^34–36^. For example, lysosomes localized SIDT2 could effectively deliver RNAs into lysosomes during RNautophagy^37^. SQSTM1 is the well-known autophagy receptor which recruits polyubiquitinated proteins to be degraded to LC3-positive autophagosomes^38,39^. In our study, we found SQSTM1 could directly interact with ARHGAP5-AS1 and recruit it to autophagosome for degradation, thus extending SQSTM1 as an RNautophagy adapter. Interestingly, recent studies have revealed the key role of SQSTM1 as a putative RNA-binding protein in aggrephagy, protein–RNA aggregate clearance by autophagy^36,38,39^. While we defined the particular sequence of ARHGAP5-AS1 is responsible for its interaction with SQSTM1, it remains unknown the molecular mechanism about dynamic regulation in SQSTM1 recognition of ARHGAP5-AS1.

Increasing evidences have indicated lncRNAs as the dominator to modulate vast majority of physiological and pathological processes^12,40^. Dysregulation of these non-coding molecules, thus, contributed to the development of many disorders such as cancers. LncRNAs are multifunctional to affect the functions and stabilities of various proteins or nuclear acids. Among them, one particular class is natural anti-sense transcripts (NATs). NATs are able to regulate the expression of their target genes both in *cis* and in *trans*^41,42^. NAT is involved in gene transcription and alternative splicing, based on RNA–DNA/RNA and RNA–protein interactions. For example, AS1DHRS4 repressed the transcription of DHRS4 gene cluster by simultaneously pairing with ongoing sense transcripts and recruiting epigenetic regulators, such as DNA and histone methyltransferases to remodel chromatin^43^. In addition, NATs can regulate the stability of various RNAs including mRNA and other non-coding RNAs, such as microRNAs by direct antisense–sense RNA interactions^44^. Herein, we identified ARHGAP5-AS1 as a head to head NAT of ARHGAP5 to regulate both the generation and stability of ARHGAP5 mRNA in the nucleus and cytoplasm, respectively.

While the mechanism for lncRNAs in chromatin remodeling has been well explored, how lncRNAs affect RNA stability in the cytoplasm remains largely unknown. We found that ARHGAP5-AS1 can recruit RNA modifying enzymes to affect RNA stability, resembling the recruitment of epigenetic modifiers to remodel the chromatin. As catalyzed by METTL3/METTL14/WTAP methyltransferase complex, N6-methyladenosine (m^6^A) is the most prevalent and conservative RNA modification in mammalian cells^45^. However, the recognition of particular RNAs to be m^6^A modified by methyltransferase complex has not been clarified. Interestingly, ARHGAP5-AS1 interacted with METTL3 and ARHGAP5 mRNA, thus facilitating m^6^A modification of ARHGAP5 mRNA in a sequence-specific manner. As a consequence, m^6^A modification could be recognized by various readers including YTHDF1-3 proteins to confer target RNAs distinct destinations, such as transportation, splicing, degradation, and protein translation^46,47^. For example, YTHDF2 binds and destabilizes m^6^A-modified RNAs. Interestingly, the interaction of ARHGAP5 mRNA with the well-established RNA stabilizer protein HuR was abrogated upon the knockdown of ARHGAP5-AS1. Meanwhile, the m^6^A modification of ARHGAP5 mRNA was also reduced. Generally, the m^6^A modification was thought to stabilize mRNA by blocking HuR binding. Indeed, certain mRNA fragments from cells with METTL3 displayed increased capability of HuR binding^48^. However, both in vitro and in vivo experiments confirmed that HuR could directly interact with m^6^A-modified mRNAs^48,49^. Therefore, spatial constraints govern m^6^A and HuR binding, indicating that HuR could directly bind to m^6^A or indirectly interact with m^6^A as one part of an m^6^A-binding ribonucleoprotein complex. Certainly, the specific m^6^A readers facilitate HuR binding warrants further investigations.

ARHGAP5 could significantly dysregulate the activity of Rho subfamily of small GTPases that plays an important role in cancer progression mainly by regulating cytoskeleton organization^50,51^. There are three major members in the Rho subfamily of small GTPases, called RhoA, RhoB, and RhoC, respectively. They are highly homologous and share same upstream regulators and downstream effectors. However, they have different roles in cancer progression. RhoA and RhoC were frequently activated in many cancers and can stimulate malignant transformation. In contrast, RhoB exhibited tumor suppressor functions by promoting cell apoptosis. Moreover, the inactivation of RhoA by αGCF2/LRRFIP1 conferred resistance to cisplatin mainly through enhancing DNA damage repair or silencing cytoskeleton/trafficking genes^52,53^. Therefore, ARHGAP5 can promote cancer progression by enhancing proliferation, migration, and invasion of various cancer cells^54–56^. Furthermore, ARHGAP5 can also facilitate cancer development independent on RhoA activation^57,58^. We presented evidence that it was upregulated in chemoresistant cancer cells and its knockdown succeeded to reverse chemoresistance, extending its tumor-promoting functions. At large, targeting ARHGAP5 and its upstream regulators might be a novel strategy to overcome chemoresistance.

In summary, ARHGAP5-AS1 is a new chemoresistance-promoting antisense lncRNA. Autophagy adaptor SQSTM1 recruited it for autophagic degradation and it was upregulated in chemoresistant cancer cells resulting from impaired autophagy. It stimulated ARHGAP5 transcription in the nucleus and stabilized ARHGAP5 mRNA in the cytoplasm by recruiting METTL3 for m^6^A modification and HuR binding of ARHGAP5 mRNA. Inhibiting either ARHGAP5-AS1 or ARHGAP5 succeeded to reverse chemoresistance (Fig. 7g). Therefore, targeting ARHGAP5-AS1/ARHGAP5 axis could be a novel strategy to the clinical management of chemoresistance.

## Supplementary information

Supplementary Files
