# Supplementary material for: Impaired autophagic degradation of lncRNA ARHGAP5-AS1 promotes chemoresistance in gastric cancer
Source: Cell Death Dis. 2019 May 16;10(6):383. doi: 10.1038/s41419-019-1585-2 (PMC6522595; doi:10.1038/s41419-019-1585-2)
Supplement: Supplementary file 1 — Supplementary Files [file 41419_2019_1585_MOESM1_ESM.pdf]

Supplementary Table 1 List of primers or siRNAs

name	application	sequence(5'-3')
ARHGAP5-AS1-F	qRT-PCR	CTCAAGAGCAAACCACCGTAC
ARHGAP5-AS1-R	qRT-PCR	ACATGTTCCTGCGAACGAG
ARHGAP5-F	qRT-PCR	GACCGACGTTGTTAGGAAGATG
ARHGAP5-R	qRT-PCR	GGGGACGAGGCTCTTTGTTT
ARHGAP5-AS1-FL-F	Overexpression	CCCAAGCTTTCTCCCACCATTGGTTCC
ARHGAP5-AS1-FL-R	Overexpression	CGGGATCCTGGAATATTGTGACAAATTT ACTGAC
ARHGAP5-AS1-S1-F	biotin-probe	CCCAAGCTTGGTCGTAACTCAAGA GCAAACC
ARHGAP5-AS1-S1-R	biotin-probe	CGGGATCCGACGGAGAAATGGACT CCTCT
ARHGAP5-AS1-S2-F	biotin-probe	CCCAAGCTTAGAGGAGTCCATTTCT CCGTC
ARHGAP5-AS1-S2-R	biotin-probe	CGGGATCCTCCTTTCTCACTGCAC CATTC
ARHGAP5-AS1-S3-F	biotin-probe	CCCAAGCTTGAATGGTGCAGTGAG AAAGGA
ARHGAP5-AS1-S3-R	biotin-probe	CGGGATCCCAACCTTGGCTAACTT CAGTACC
ARHGAP5-AS1-S4-F	biotin-probe	CCCAAGCTTGGTACTGAAGTTAGC CAAGGTTG
ARHGAP5-AS1-S4-R	biotin-probe	CGGGATCCGTGGCTCGAATAAAGC ATTTTAG
ARHGAP5-AS1-S5-F	biotin-probe	CCCAAGCTTCTAAAATGCTTTATTC GAGCCACT
ARHGAP5-AS1-S5-R	biotin-probe	CGGGATCCCTTTTTATATGTTTACT TCGGGTATTCTTAAG
ARHGAP5-promoter-P1-F	ChIP	GGTTGGACCACCCACCAAA
ARHGAP5-promoter-P1-R	ChIP	GCCACTCCCTTGCATCTGAA
ARHGAP5-promoter-P2-F	ChIP	TTGCTCATTCTTTTATCTGCTGGC
ARHGAP5-promoter-P2-R	ChIP	CTGGGCTTTGGGTGTGTTTC
ARHGAP5-promoter-P3-F	ChIP	TCGATCCACGTCTTGGCTTC
ARHGAP5-promoter-P3-R	ChIP	GAGGATCGCTCGCCAACTAC
ARHGAP5-promoter-P4-F	ChIP	GAAAAATACTCACGCCAATGGGTA

ARHGAP5-promoter-P4-R	ChIP	GAGGGAGGGGGACGAGC
ARHGAP5-promoter-P5-F	ChIP	CGGGAGAGATGCTGAGGAA
ARHGAP5-promoter-P5-R	ChIP	ACTCTCTTCTCATGTCTCTACATTC
ARHGAP5-AS1-siRNA-1	Knock down	UUGCUCUUGAGUUACGACCTT
ARHGAP5-AS1-siRNA-2	Knock down	AAUGGACUCCUCUUCUCGCTT
ARHGAP5-siRNA-1	Knock down	AAGCCUAGCUGAUCAGUGCTT
ARHGAP5-siRNA-2	Knock down	AUACCUCUUUGCUAUCAGCTT
HuR-siRNA-1	Knock down	ATGTGAAAGTGATTCGTGA
HuR-siRNA-2	Knock down	GCTTATTCGGGATAAAGTA
SQSTM1-siRNA-1	Knock down	AUUGUCAAUUCCUCGUCACTT
SQSTM1-siRNA-2	Knock down	UGAACAGUUAUCCGACUCCTT
METTL3-siRNA-1	Knock down	UAGUGAACAUACUUGCAGGTT
METTL3-siRNA-2	Knock down	AUACUGACGUCCAGGUAGCTT
METTL14-siRNA-1	Knock down	AUCUGUCUCUCCUUCAUCCTT
METTL14-siRNA-2	Knock down	UAAAUGAUCGAGGUGCUGCTT
WTAP-siRNA-1	Knock down	UAAGCAUUCGACACUUCGCTT
WTAP-siRNA-2	Knock down	AAUGGUACUCUGCAUACCCTT

Supplementary Figures and Figure legends

Supplementary Figure 1

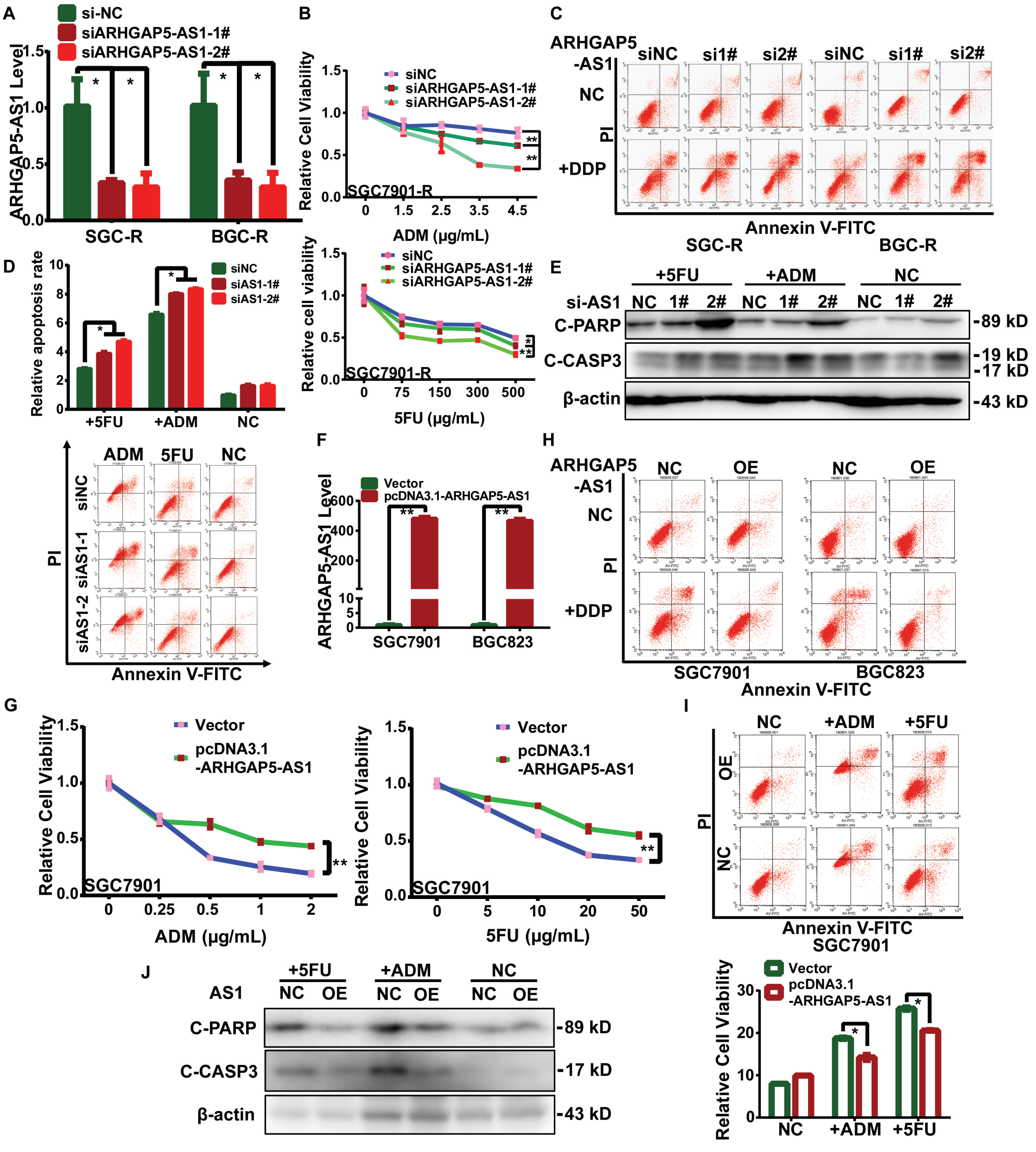

Supplemental Figure 1. ARHGAP5-AS1 was upregulated to promote chemoresistance in cancer cells. A, The level of ARHGAP5-AS1 in resistant cells before and after siRNA transfection were measured using qRT-PCR. Data were presented as the mean ± SD, n = 3. *p< 0.05 (*t* test). **B,** The effect of ARHGAP5-AS1 knockdown on relative cell viability of SGC-R cells before and after ADM or 5FU treatment for 36h were detected using MTS assay. Experiments were all repeated three times and data shown with the most

representative one. *p< 0.05 (*t* test). **C,** The raw flow cytometry apoptosis signal of resistant cells after transfecting siNC or ARHGAP5-AS1 siRNAs and DDP (5μg/mL) treatment for 36h. The apoptosis of SGC-R cells with or without ARHGAP5-AS1 knockdown in the presence of ADM (5μg/mL) or 5FU (300μg/mL) for 36h were measured using flow cytometry (**D**) or Western Blotting of PARP1 cleavage (**E**). **F,** The level of ARHGAP5-AS1 in sensitive cells before and after the plasmid transfection were measured using qRT-PCR. **G,** The effect of ARHGAP5-AS1 overexpression on viability of SGC7901 cells with or without ADM or 5-FU treatment for 24h were detected using MTS assay. Experiments were all repeated three times and data shown with the most representative one. **p< 0.01 (*t* test). **H,** The raw flow cytometry apoptosis signal of sensitive cells after transfecting empty vector or pcDNA3.1-ARHGAP5-AS1 and DDP (2μg/mL) treatment for 24h. ARHGAP5-AS1 was overexpressed in SGC7901 cells and apoptosis induced by ADM (1μg/mL) or 5-FU (50μg/mL) were measured using flow cytometry (**I**) or Western Blotting (**J**). Experiments were all repeated three times and the most representative results were shown.

Supplementary Figure 2

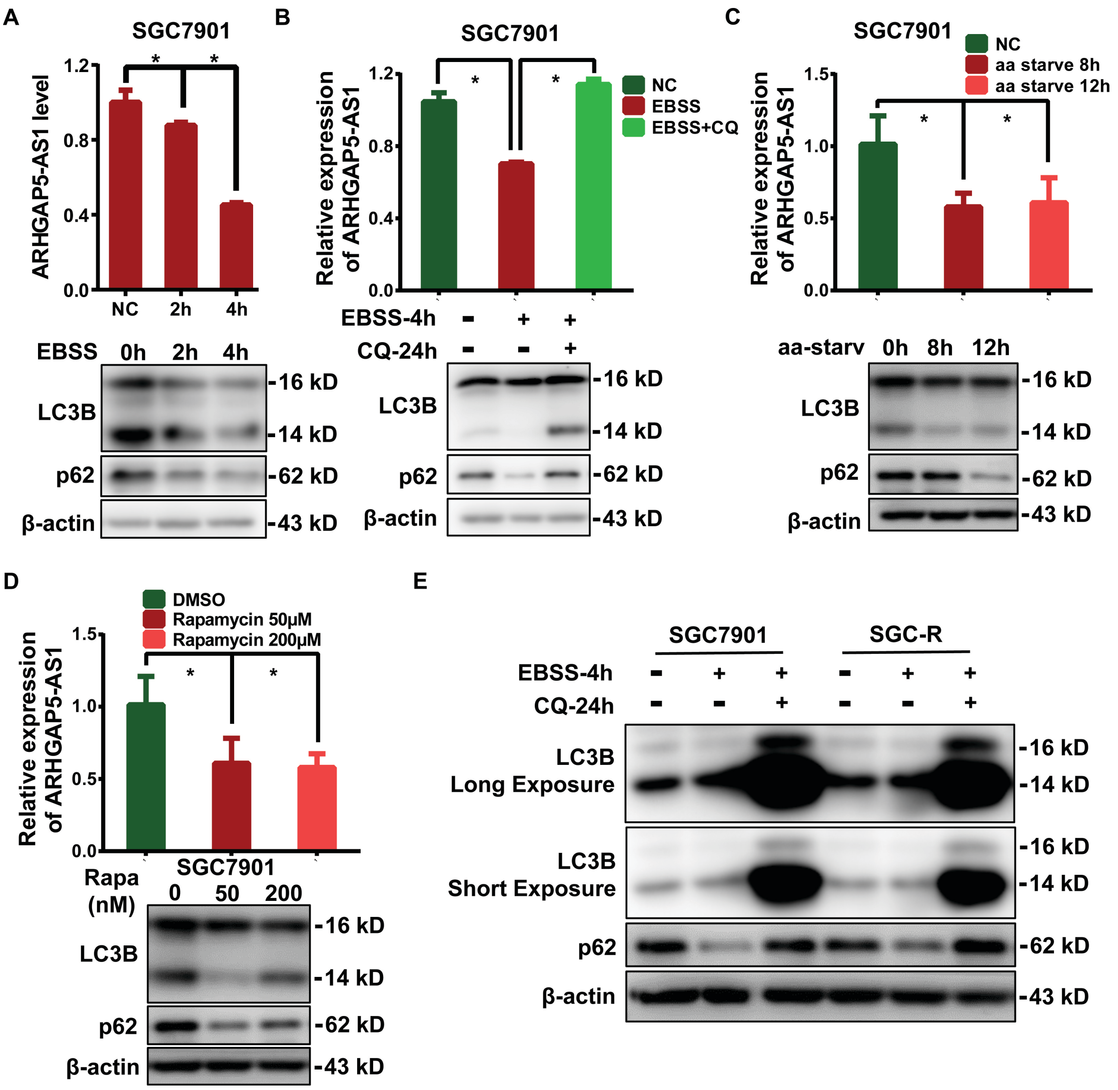

Supplemental Figure 2. Autophagy-dependent degradation of ARHGAP5-AS1. A, ARHGAP5-AS1 levels in SGC7901 cells with EBSS treatment for 2h or 4h were determined by qRT-PCR (Top panel). Autophagy induction was confirmed by Western Blotting (Bottom panel). **B,** ARHGAP5-AS1 expression in SGC7901 cells treated with CQ (50μM, 24h) and EBSS (4h) were detected by qRT-PCR (Top panel). Autophagy was assessed using Western Blotting (Bottom panel). **C,** ARHGAP5-AS1 expression in SGC7901 cells with amino acid starvation were detected using qRT-PCR (Top panel). Autophagy was measured using Western Blotting

(Bottom panel). **D,** ARHGAP5-AS1 expression in SGC7901 cells treated with rapamycin (48h) were detected by qRT-PCR (Top panel). Autophagy was assessed by Western Blotting (Bottom panel). **E,** Autophagy in SGC7901 and SGC-R cells treated with EBSS (4h) and/or CQ (50μM, 24h) were analyzed by Western blotting.

Supplementary Figure 3

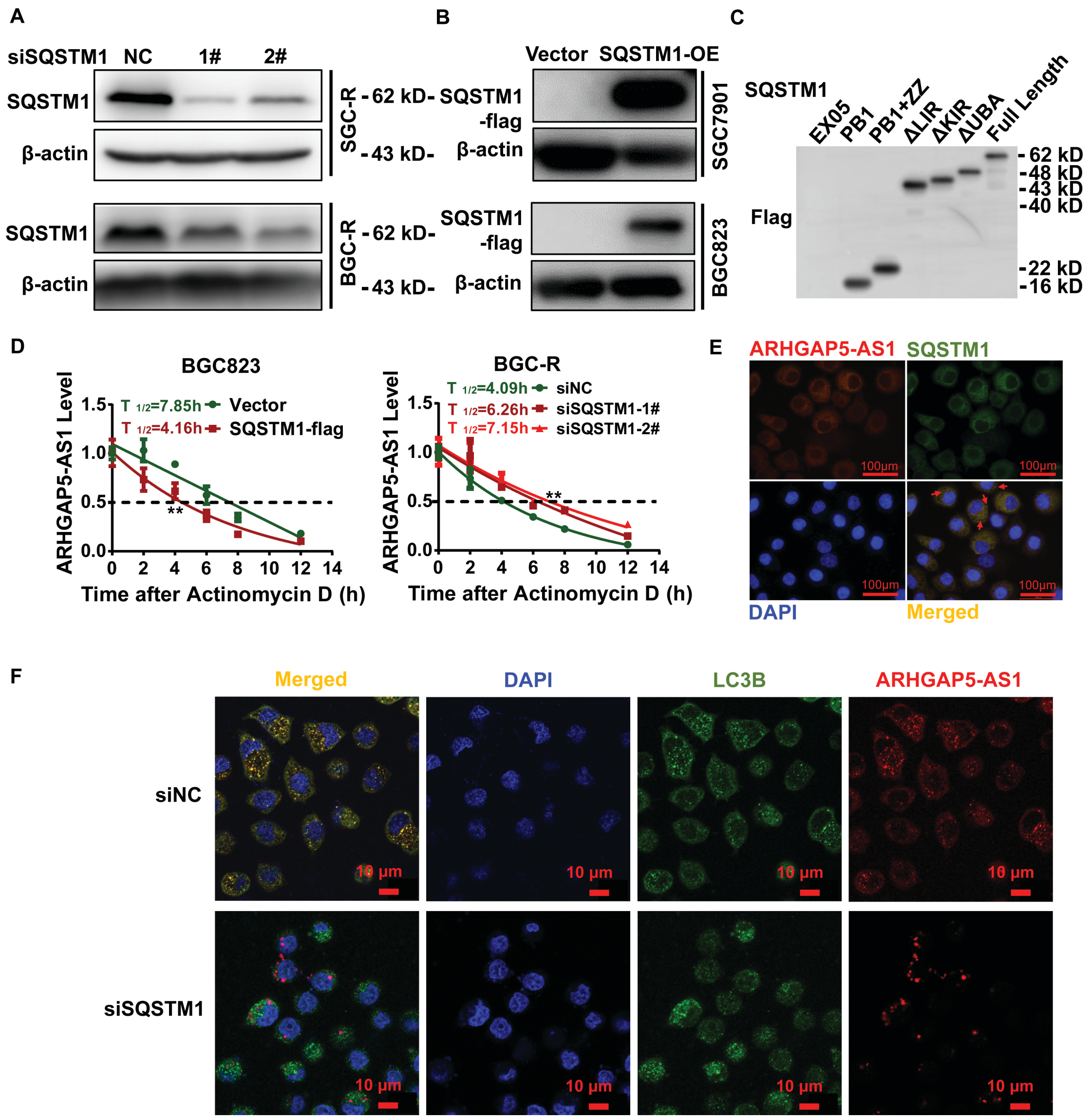

Supplemental Figure 3. SQSTM1 recruited ARHGAP5-AS1 for autophagic degradation. A, SQSTM1 expression in SGC-R cells transfected with siNC or siSQSTM1-siRNAs were determined by Western Blotting. **B,** SQSTM1

expression in SGC7901 cells with SQSTM1 overexpression were determined by Western Blotting. **C,** Expression of various SQSTM1 constructions were confirmed by Western Blotting. **D,** Half-life of ARHGAP5-AS1 in BGC823 and BGC-R cells with SQSTM1 overexpression or knockdown were analyzed by qRT-PCR. **E,** Detection of ARHGAP5-AS/SQSTM1 colocalization in SGC-R cells using combined FISH and IFC assay. Red: ARHGAP5-AS1; Green: SQSTM1; Blue: DAPI. (original magnification, 100X). Scale bar: 100 μm. **F,** The colocalization detection of ARHGAP5-AS/LC3B in SGC-R cells treated as indicated with combined FISH and IFC assay. Red: ARHGAP5-AS1; Green: LC3B; Blue: DAPI. (original magnification, 00X). Scale bar: 10μm.

Supplementary Figure 4

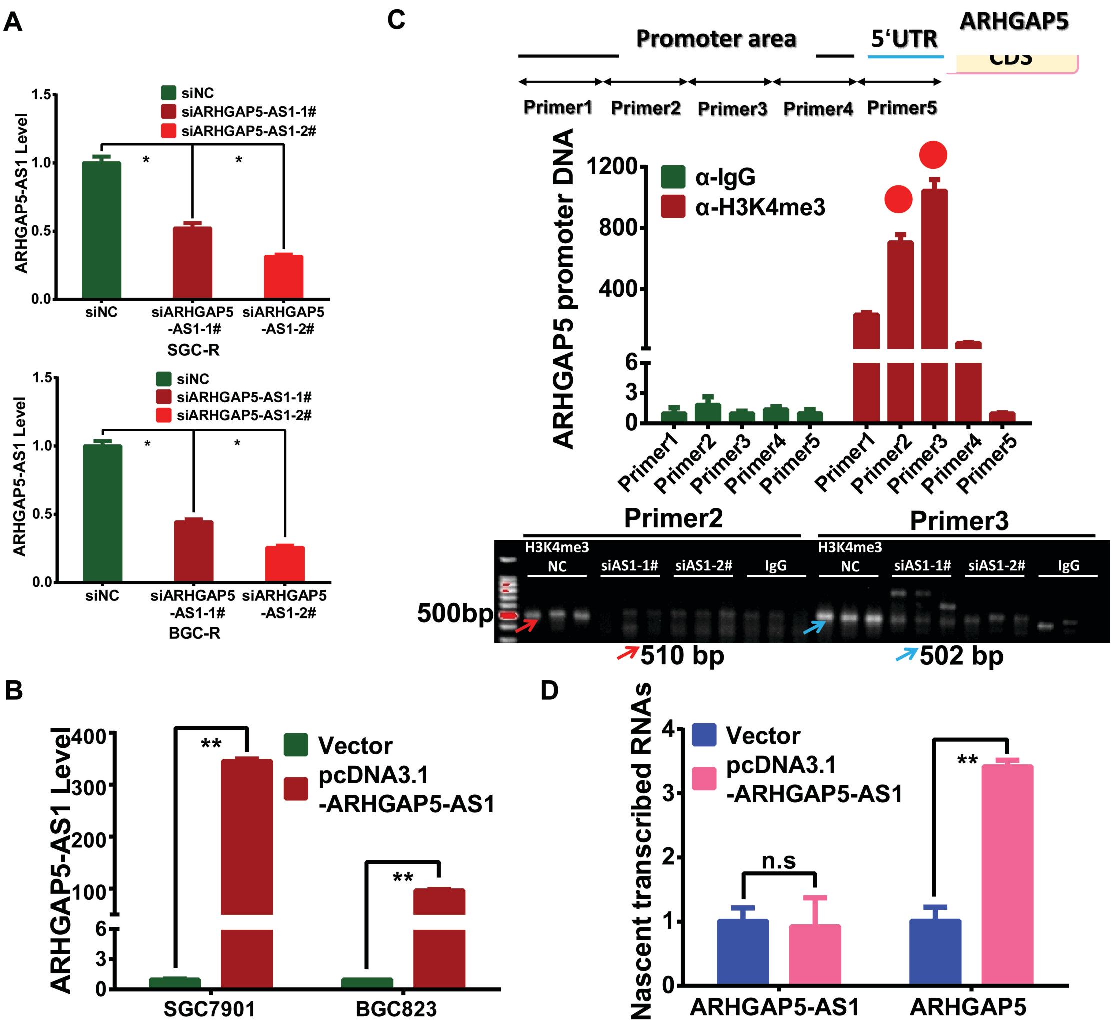

Supplementary Figure 4. ARHGAP5-AS1 stimulated ARHGAP5 transcription. A, ARHGAP5-AS1 level in resistant cells after transfecting siNC or ARHGAP5-AS1 siRNAs were assessed by qRT-PCR. **B,** ARHGAP5-AS1 expression in SGC7901 and BGC823 cells after transfection were validated by qRT-PCR. **C,** The schematic representation of primers used for ChIP-PCR analysis of H3K4me3-occupied ARHGAP5 promoter (Top panel, the red circle indicated the core promoter area; Bottom panel, Agarose gel electrophoresis of the PCR products). **D,** The transcription efficiency of ARHGAP5 mRNA after overexpressing ARHGAP5-AS1 was determined using Click it assay.

Supplementary Figure 5

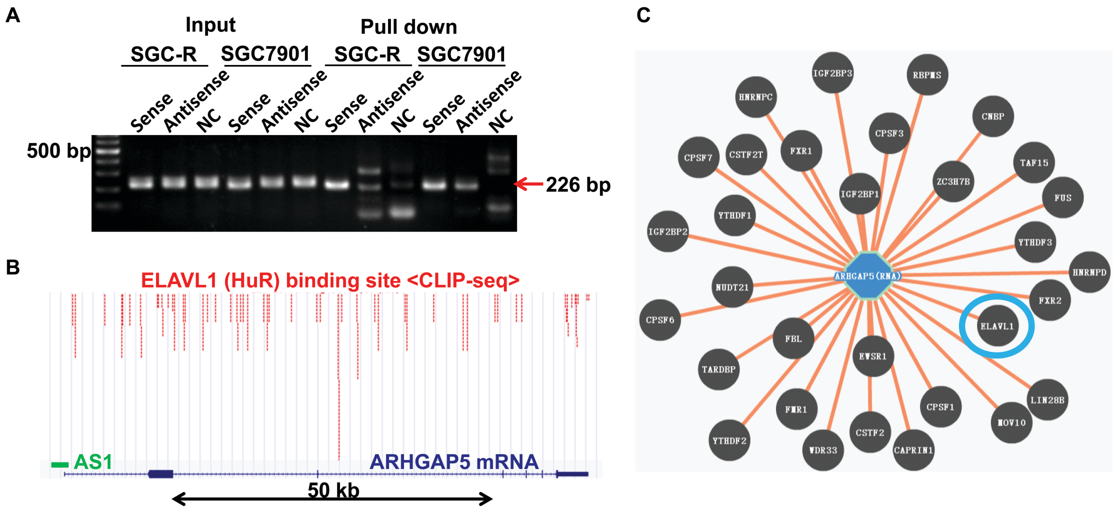

Supplemental Figure 5. ARHGAP5-AS1 stabilized ARHGAP5 mRNA in the cytoplasm. A, Agarose gel electrophoresis of PCR products from biotin pull down assay in Figure 5C. **B,** The visualization of HuR binding site in ARHGAP5 mRNA using UCSC browser. Data was derived from CLIP-seq of GEO datasets. **C,** The interaction of ARHGAP5 mRNA with HuR was predicted using StarBase and catPARID assessment tools.

Supplementary Figure 6

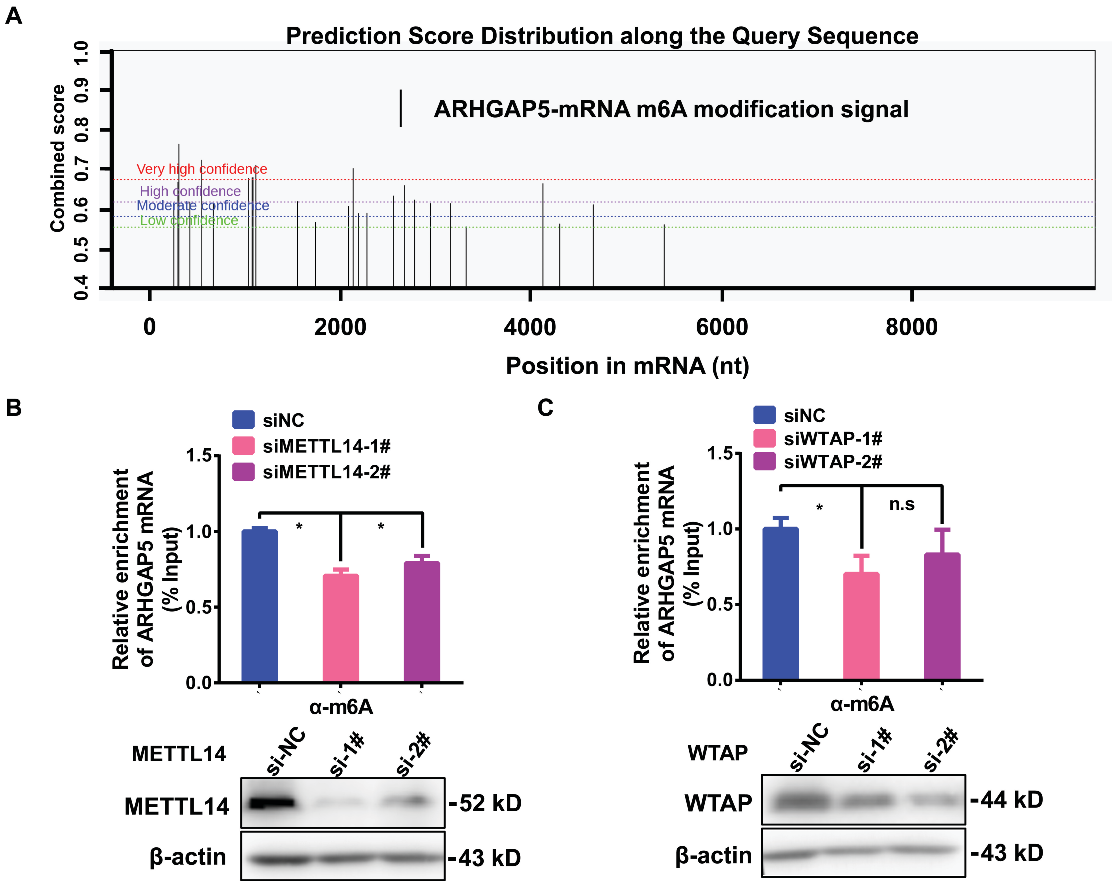

Supplemental Figure 6. ARHGAP5-AS1 recruited METTL3 for m6A modification of ARHGAP5 mRNA. A, Prediction of m6A modification in ARHGAP5 mRNA using SRAMP assessment tool. **B-C,** The effect of METTL14 or WTAP downregulation on m6A modification of ARHGAP5 mRNA were assessed by RIP assay.

Supplementary Figure 7

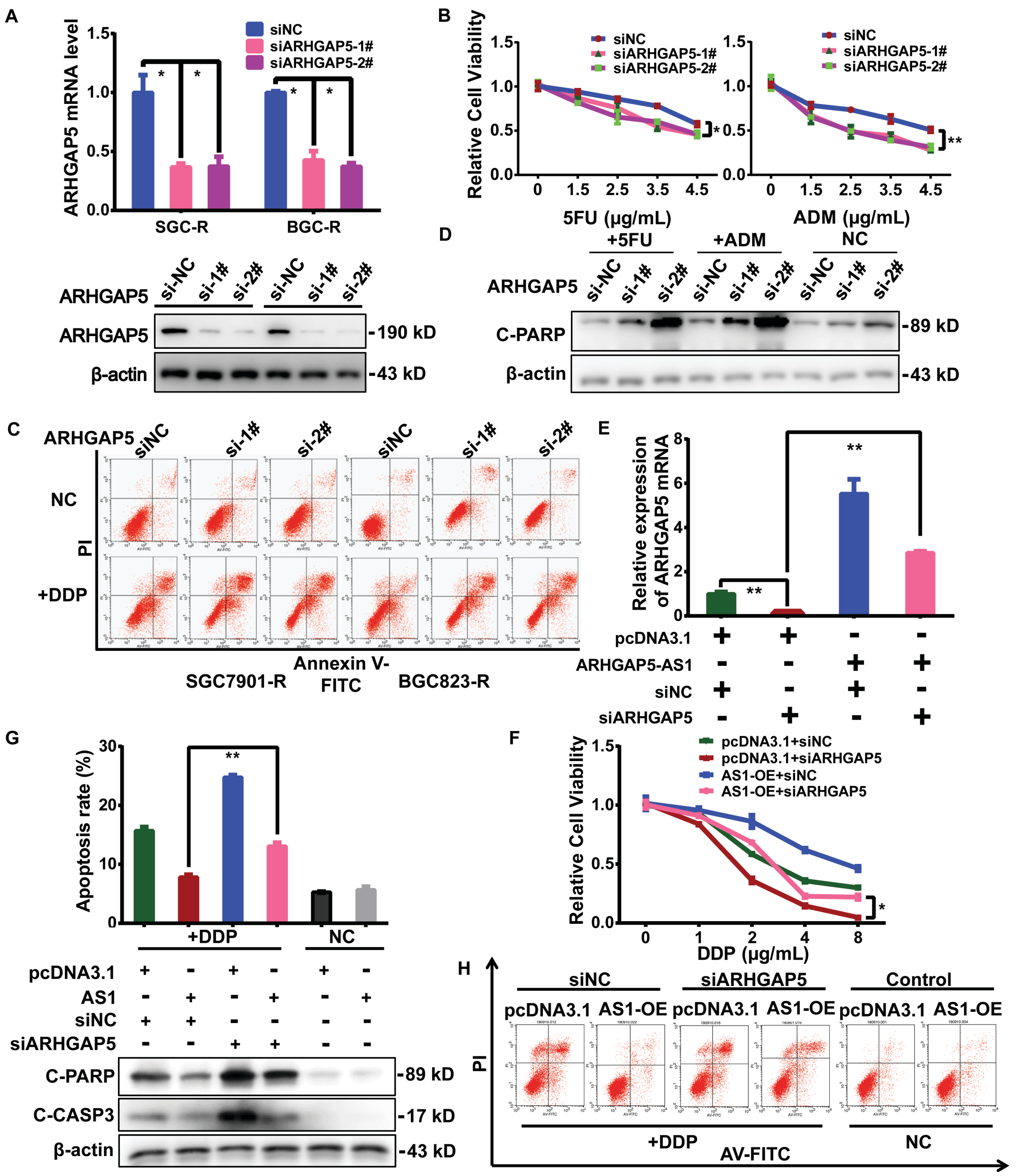

Supplemental Figure 7. ARHGAP5 promoted chemoresistance in cancer cells. A, The level of ARHGAP5 mRNA (Top panel) and protein (Bottom panel) in resistant cells after knocking ARHGAP5 down were measured using qRT-PCR and Western Blotting, respectively. **B,** Effects of ARHGAP5 knockdown on viability of SGC-R cell before and after ADM or 5FU treatment for 36h were detected using MTS assay. **C,** The raw flow cytometry signal of

resistant cells after transfecting siNC or ARHGAP5 siRNAs and DDP (5μg/mL) treatment for 36h. **D,** ARHGAP5 was knocked down in SGC-R cells and the apoptosis before or after ADM (5μg/mL) or 5FU (300μg/mL) treatment for 36h were measured using Western Blotting. **E,** ARHGAP5 mRNA in SGC7901 cells treated as indicated were validated using qRT-PCR. **F,** Viability of SGC7901 cells treated as indicated were detected using MTS assay. **G,** Apoptosis of SGC7901 cells treated as indicated were detected by flow cytometry (Top panel) and Western Blotting (Bottom panel), respectively. **H.** The raw flow cytometry signal of DDP (2μg/mL, 24h) treated SGC7901 cells after transfecting ARHGAP5-AS1 and ARHGAP5 siRNAs.
